# Clicked and long spaced galactosyl- and lactosylcalix[4]arenes: new multivalent galectin-3 ligands

**DOI:** 10.3762/bjoc.10.175

**Published:** 2014-07-23

**Authors:** Silvia Bernardi, Paola Fezzardi, Gabriele Rispoli, Stefania E Sestito, Francesco Peri, Francesco Sansone, Alessandro Casnati

**Affiliations:** 1 Dipartimento di Chimica, Università degli Studi di Parma, Parco Area delle Scienze 17/a, 43124 Parma, Italy; 2 Dipartimento di Biotecnologie e Bioscienze, Università degli Studi di Milano-Bicocca, Piazza della Scienza 2, 20126 Milano, Italy

**Keywords:** glycocalixarenes, cluster glycoside effect, multivalency, click chemistry, surface plasmon resonance

## Abstract

Four novel calix[4]arene-based glycoclusters were synthesized by conjugating the saccharide units to the macrocyclic scaffold using the CuAAC reaction and using long and hydrophilic ethylene glycol spacers. Initially, two galactosylcalix[4]arenes were prepared starting from saccharide units and calixarene cores which differ in the relative dispositions of the alkyne and azido groups. Once the most convenient synthetic pathway was selected, two further lactosylcalix[4]arenes were obtained, one in the cone, the other one in the 1,3-alternate structure. Preliminary studies of the interactions of these novel glycocalixarenes with galectin-3 were carried out by using a lectin-functionalized chip and surface plasmon resonance. These studies indicate a higher affinity of lactosyl- over galactosylcalixarenes. Furthermore, we confirmed that in case of this specific lectin binding the presentation of lactose units on a cone calixarene is highly preferred with respect to its isomeric form in the 1,3-alternate structure.

## Introduction

**Figure 1:**
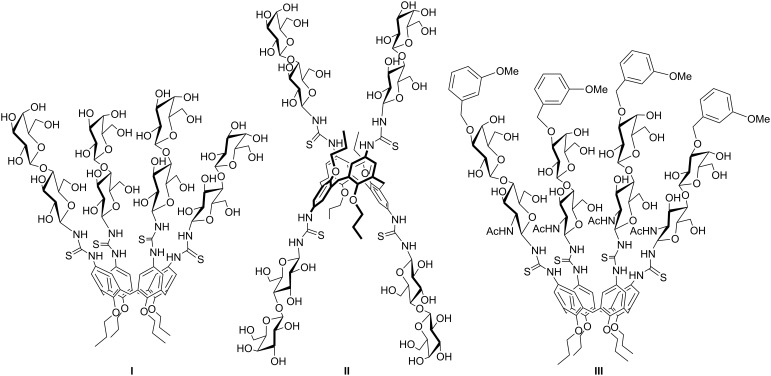
Lactosylthioureidocalix[4]arenes **I–III** used to inhibit Gal-3 [20–21].

Lectins are carbohydrate-binding proteins (CBP) [1–3] without any catalytic or immunogenic activity. In the latest decades, they attracted an increasing interest due to their involvement in a series of fundamental biological processes such as cell adhesion, cell activation, cell growth, differentiation and apoptosis. Among different families of lectins, the ones showing a selectivity for β-D-galactoside and β-D-galactose-terminating oligosaccharides are called galectins and play important roles in a series of pathological events such as inflammation, fibrosis, heart diseases and cancer [4–5]. The role of one member of this family in particular, namely galectin-3 (Gal-3), has been intensively investigated lately and it was shown that it is deeply involved in cancer metastasis and migration. Based on these findings and with the aim to inhibit its activity and to target it for therapeutic or diagnostic purposes, Gal-3 became a rather important target in medicine. Remarkably interesting is the intra-family selectivity and, especially, the ability to block Gal-3 but not Gal-1. Gal-1, in fact, can act as anti-inflammatory agent, while Gal-3 has a pro-inflammatory activity [6]. Furthermore, Gal-3 can act as a competitive inhibitor against Gal-1 which, on the other side, induces anoikis of tumor cells [7–8]. Glycocalixarenes [9–12], calixarenes [13–15] adorned with carbohydrates at the upper and/or at the lower rims, have been demonstrated to be efficient multivalent ligands for a series of pathological lectins. For instance, cholera toxin is bound rather efficiently by calix[4]arene [16] and calix[5]arene [17] derivatives, while examples of *Pseudomonas aeruginosa* LecB binding were reported with galactosylcalixarenes blocked in different conformations [18–19]. A few years ago we [20–21] reported about the synthesis and inhibitory properties of a small library of lactosylthioureidocalixarenes and found that the cone derivatives **I** and **III** (Figure 1) were able to efficiently inhibit the adhesion of Gal-3 to tumor cells in vitro, but not that of galectin-1 [22].

**Figure 2:**
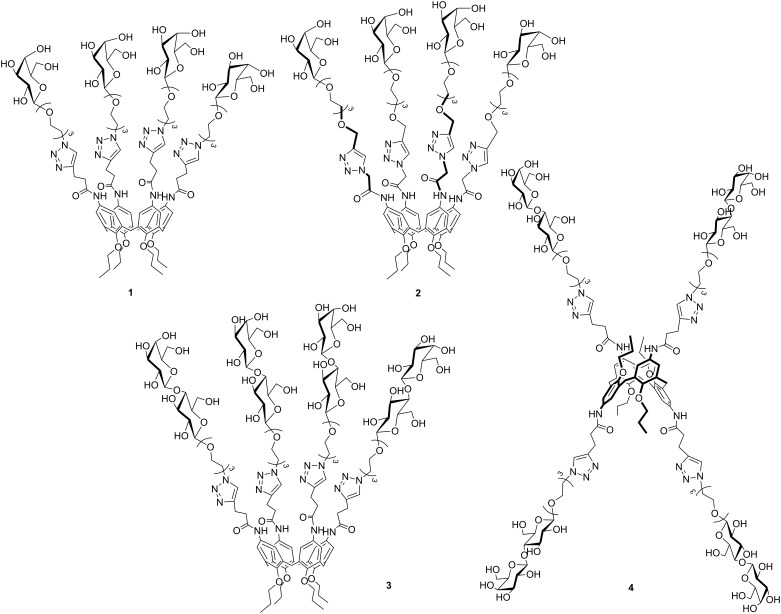
The new glycocalix[4]arenes **1–4** synthesized in this study.

The opposite behavior was observed for the 1,3-alternate derivative **II**, able to inhibit Gal-1 but not Gal-3. On the basis of these findings, we herein report the synthesis of a new subfamily of galactosyl- and lactosylcalix[4]arenes **1–4** (Figure 2) which are characterized by long hydrophilic spacers between the glycosyl units and the multivalent calixarene scaffold. We also report on preliminary studies of the interaction of the novel subfamily of galactosyl- and lactosylcalix[4]arenes with Gal-3 by surface plasmon resonance (SPR).

## Results and Discussion

### Synthesis of the glycocalixarenes

“Click Chemistry” [23] reactions are extensively used to conjugate (oligo)saccharides to macrocyclic structures due to the mild conditions and the high yields [24]. For the synthesis of glycocalixarenes the amino–isothiocyanate condensation [25–30] or the 1,3-dipolar cycloaddition have been widely studied in their scope and limitations [10,31]. In particular, the Huisgen cycloaddition reaction was first applied to a calixarene in 2000 by Santoyo-González [32]. Later on, Marra et al. [33] demonstrated that the copper-catalyzed azide–alkyne cycloaddition (CuAAC) [34–35] at room temperature could afford divalent and tetravalent glycocalixarenes in very high yields and regioselectivity. Following these studies, a wide series of other examples appeared in the literature [18,36–39] also exploiting the use of microwaves, ionic liquids and protected or even deprotected [17] saccharides. Usually, either the strategy of reacting an alkynylated-saccharide with a polyazide calixarene (dipolarophile-on-the-sugar) or an azido-sugar and a polyalkynocalixarene (dipolarophile-on-the-calix) work smoothly [33]. However, a sort of autocatalytic effect was evidenced in the case of the reaction between a 1-ethynyl-C-glycoside with a tetraazidocalix[4]arene (dipolarophile-on-the sugar strategy). It was suggested by the authors that the first intermolecular reaction, leading to a Cu-triazolide adduct, allows the copper ion to coordinate an ethynyl glycoside, thus entailing an intramolecular CuAAC reaction with an adjacent azido-arm [37].

**Scheme 1:**
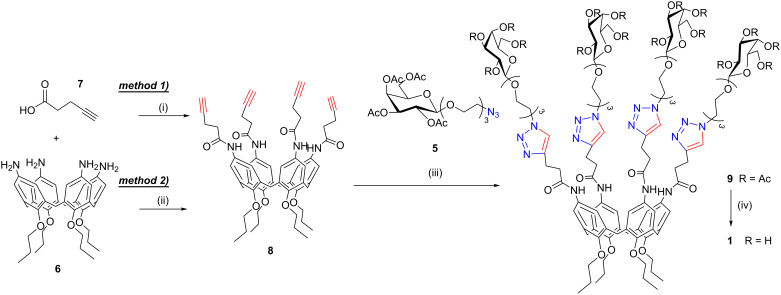
Synthesis of the cone galactosylcalix[4]arenes **1**. Reaction conditions: (i) DCC, DMAP, CH_2_Cl_2_, under reflux, 5 h, 44%; (ii) EDC, CH_2_Cl_2_/py (7:3), rt, 18 h, 66%; (iii) CuSO_4_·5H_2_O, Na-ascorbate, DMF/H_2_O, μW (150 W), 80 °C, 20 min, 83%; (iv) CH_3_ONa, CH_3_OH, rt, 1 h – IR120/H^+^, rt, 30 min, quantitative.

Firstly, we decided to evaluate the effectiveness of the two approaches dipolarophile-on-the-calix and dipolarophile-on-the-sugar by using a galactose and cone calixarene scaffolds. This investigation was carried out with the idea to extend the study to stronger ligating units for galectins such as lactose and different calixarene structures, also including the 1,3-alternate isomer. The first route explored (dipolarophile-on-the-calix) was applied to the preparation of the multivalent compound **1**, which could be synthesized by exploiting a convergent synthetic approach. This approach was based on the connection, by CuAAC reaction, of the azido-terminating tetraacetylgalactose **5** [40–41] to calix[4]arene **8** decorated at the upper rim with alkyne terminating chains (Scheme 1).

In order to introduce the alkyne units at the upper rim of the macrocycle, we decided to exploit the easily available and highly versatile *p*-aminocalixarene **6** prepared according to literature procedures [25]. The coupling reaction between amino-calix[4]arene **6** and 4-pentynoic acid (**7**) in the presence of dicyclohexylcarbodiimide (DCC) led to compound **8** in 44% yield. Due to the concurrent formation of dicyclohexylurea (DCU), several purification steps were necessary to obtain pure calix[4]arene **8**. The use of 1-ethyl-3-(3-dimethylaminopropyl)carbodiimide (EDC) as an alternative coupling agent allowed us to isolate pure compound **8** in a more straightforward way and higher yield (66%). Any attempts to connect the alkyne functionality in closer proximity to the calixarene core by decreasing the number of carbon atoms between the carboxylic group and the triple bond did not give fruitful results. Reactions between amino-calix[4]arene **6** and propiolic acid were carried out with a variety of coupling agents. In the presence of DCC the tetra-condensation product was only obtained in very low yields. Furthermore, it was not possible to purely isolate it from the crude reaction mixture due to the high amount of byproducts formed during the reaction. The CuAAC reaction between the tetraalkyne calix[4]arene **8** and azido-galactoside **5** to give glycocluster **9** (83% yield) was carried out in DMF and H_2_O with CuSO_4_ and sodium ascorbate following a microwave-assisted procedure (20 min, 150 W, 80 °C). No partially functionalized compounds or other byproducts were detected in the crude mixtures.

**Scheme 2:**
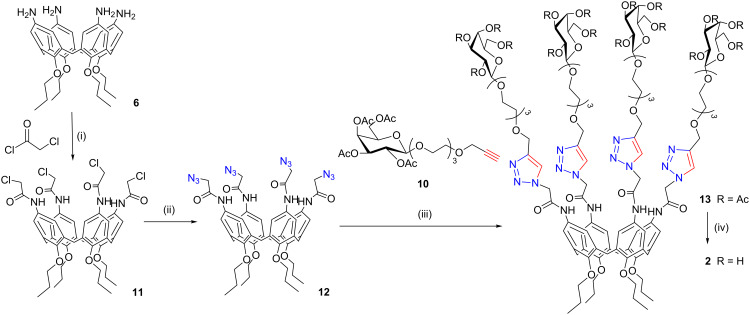
Synthesis of the cone galactosylcalix[4]arenes **2**. Reaction conditions: (i) DIPEA, CH_2_Cl_2_, rt, 6 h, 55%; (ii) NaN_3_, CH_3_OH/DMF, reflux, 1 h, 75%; (iii) CuSO_4_·5H_2_O, Na-ascorbate, DMF/H_2_O, μW (150 W), 80 °C, 20 min, 81%; (iv) CH_3_ONa, CH_3_OH, rt, 1 h – IR120/H^+^, rt, 30 min, quantitative.

The second strategy studied (dipolarophile-on-the-sugar) also exploits a convergent approach, but in this case an alkyne-functionalized galactose **10** was prepared according to literature [42], so that it reacts with calixarene **12**, which was previously functionalized with azido terminating arms (Scheme 2). This latter compound was synthesized in two steps starting again from tetraamino derivative **6**. In the first step compound **6** was treated with chloroacetyl chloride to give the α-choloroacetamido compound **11** [43]. Subsequent substitution of the chlorine ions with azide groups led to the formation of the tetraazidocalixarene derivative **12** (1 h, 75% yield).

The CuAAC conjugation reaction was carried out following exactly the same procedure as for compound **9** and allowed the isolation of **13** in very high yields (81%). Glycoconjugates **9** and **13** were fully characterized by ^1^H and ^13^C NMR spectroscopy, which displayed the disappearance of the alkyne protons and the appearance of the typical broad signal of 1,4-disubstituted triazole protons at 7.75–7.85 ppm (CD_3_OD/CDCl_3_). ESIMS (+) analyses showed peaks for the [M + 2Na]^2+^ and [M + 3Na]^3+^ adducts, which indicates the conjugation of all four macrocycle arms to the saccharide units. On the basis of the comparison between the efficiency of the conjugation steps bringing to glycoconjugates **9** and **13** (yields >80% in both cases) and contrary to the observation by Marra et al. [37], we could not collect any evidence for an autocatalytic effect in the dipolarophile-on-the-sugar approach [44]. The deprotection of compounds **9** and **13** from the acetyl groups was carried out by a transesterification reaction in the presence of CH_3_ONa in CH_3_OH at room temperature according to the standard Zemplén procedure. Complete deacetylation was achieved in 1 hour, as confirmed by NMR and ESIMS(+) spectra of compounds **1** and **2**. It is noteworthy that while compound **1** exhibited a high stability under Zemplén conditions even if the reaction was continued overnight, compound **2** started to decompose after 18 hours. ESIMS profiles showed the presence of products originating from a cleavage at the amide bond with a loss of the entire glycosylated chain and the formation of an amine group at the upper rim of the calixarene. For this reason and on the basis of the synthetic availability of intermediates, we decided to privilege the dipolarophile-on-the-calix route to synthesize the triazole-containing lactosylcalixarenes **3** and **4**.

We first attempted to prepare the lactoside derivative **14** by reacting peracetylated-lactose with 2-(2-(2-chloroethoxy)ethoxy)ethanol in the presence of BF_3_·Et_2_O [45]. However, we could only obtain a mixture of α and β-anomers (α/β ratio 2:3), which is very difficult to separate by flash chromatographic methods. On the other hand, the recently reported glycosylation reactions of lactose peracetate exploiting SnCl_4_ and CF_3_CO_2_Ag as promoters [46] gave compound **14** mainly as a β-anomer (α/β ratio 1:4) in 74% isolated yield.

**Scheme 3:**
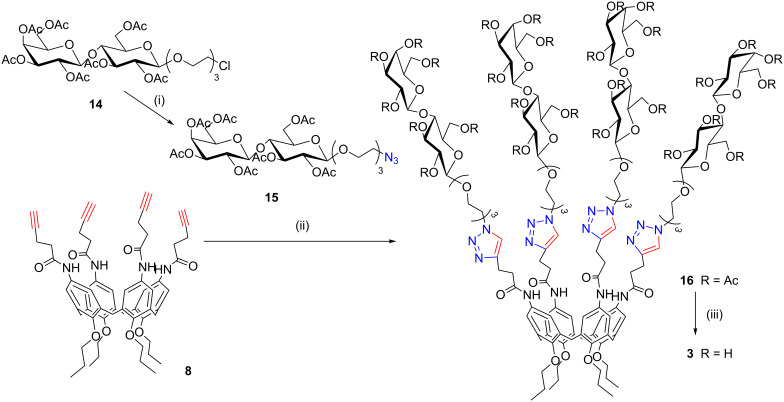
Synthesis of the cone lactosylcalix[4]arenes **3**. Reaction conditions: (i) NaN_3_, *n*-Bu_4_NI, DMF, 90 °C, 24 h, 60%; (ii) CuSO_4_·5H_2_O, Na-ascorbate, DMF/H_2_O, μW (150 W), 80 °C, 40 min, 46%; (iii) CH_3_ONa, CH_3_OH, rt, 2 h – IR120/H^+^, rt, 30 min, 73%.

**Scheme 4:**
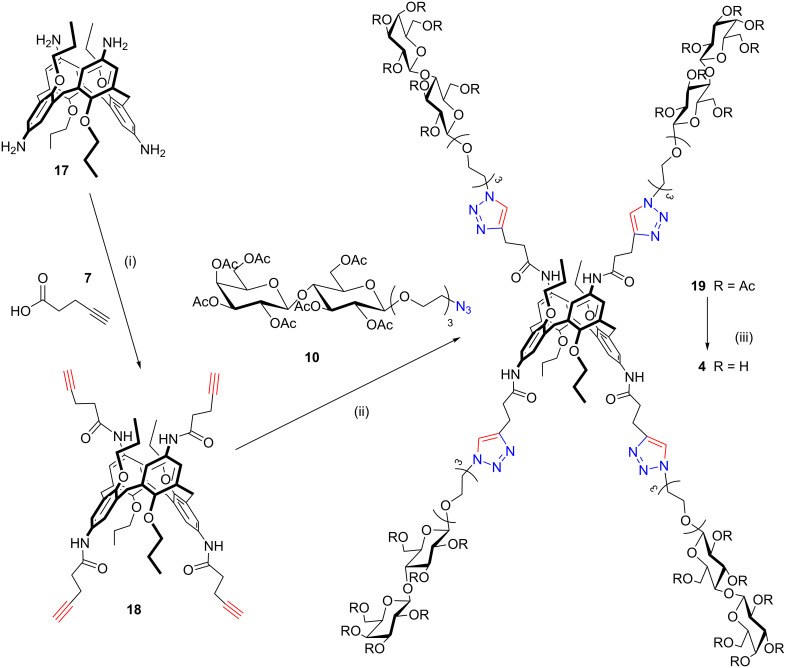
Synthesis of the 1,3-alternate lactosylcalix[4]arenes **4**. Reaction conditions: (i) EDC, CH_2_Cl_2_/py (7:3), rt, 18 h, 65%; (ii) CuSO_4_·5H_2_O, Na-ascorbate, DMF/H_2_O, μW (150 W), 80 °C, 40 min, 46%; (iii) CH_3_ONa, CH_3_OH, rt, 2 h – IR120/H^+^, rt, 30 min, 71%.

The subsequent substitution reaction of chloride with NaN_3_ (Scheme 3) led to the corresponding azido derivative **15**, which was used to “click” both the cone (**8**) and 1,3-alternate (**18**) pentynoic amides. Compound **18** was obtained from the corresponding 1,3-alternate *p*-aminocalix[4]arene **17** [47] by a reaction with EDC in CH_2_Cl_2_ and pyridine 7:3 as previously described for compound **8**. The CuAAC “click” reaction was carried out as previously described for the galacto-clusters **9** and **13** and afforded the cone calix[4]arene **16** (Scheme 3) and 1,3-alternate calix[4]arene **19** (Scheme 4) in 46% isolated yield. Microwave irradiation (150 W, 80 °C) facilitated the complete tetra-functionalization in only 40 minutes. Subsequent deacetylation with the Zemplén method led to target compounds **3** and **4**, both of which were characterized by 1D and 2D NMR techniques and ESIMS analyses.

### Gal-3/glycocalixarenes interaction studies by SPR

**Figure 3:**
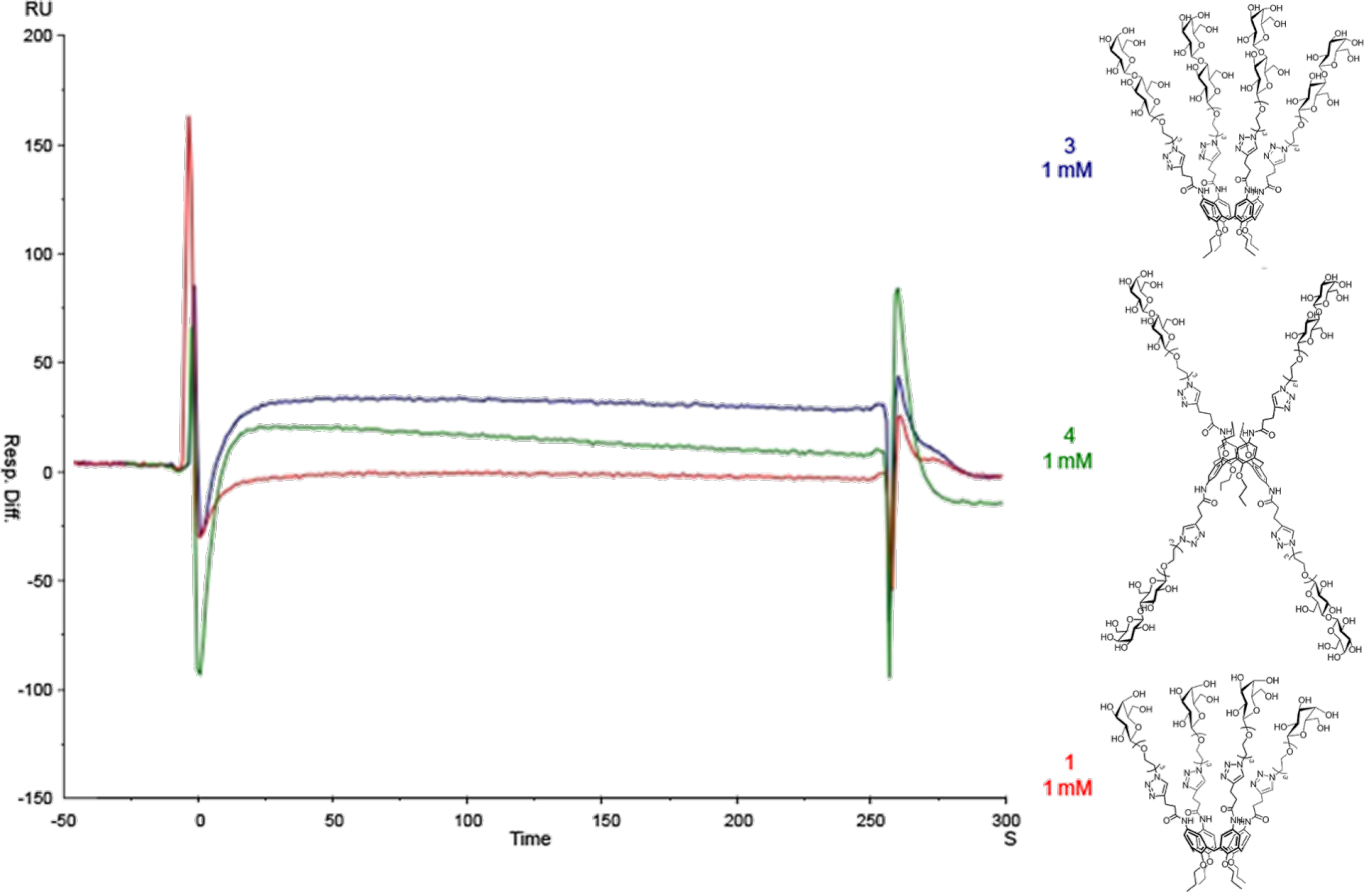
SPR sensorgrams of binding experiment between immobilized Gal-3 and glycocalixarenes **1**, **3** and **4**.

His_6_-tagged full-length Gal-3 was expressed in *E. coli* BL21 and purified on IMAC (immobilized metal ion affinity chromatography) columns. Purified protein was characterized by SDS-PAGE electrophoresis, circular dichroism (CD), and MS/MS analysis upon digestion on trypsin gel (see Figure S15, Supporting Information File 1). A preliminary evaluation of the interaction between the glycocalixarenes **1**, **3**, **4** and Gal-3 was obtained by SPR analysis by using a His-tagged Gal-3 immobilized on a Ni-NTA chip and the glycocalixarenes in solution. This approach differs from other SPR studies of the calixarene–galectin interaction with the protein in solution [38] and is tailored to have the immobilized protein properly oriented for the interaction with ligands. The sensorgrams shown in Figure 3 were obtained by fluxing an 1 mM solution of calixarenes over the protein-coated chip.

**Figure 4:**
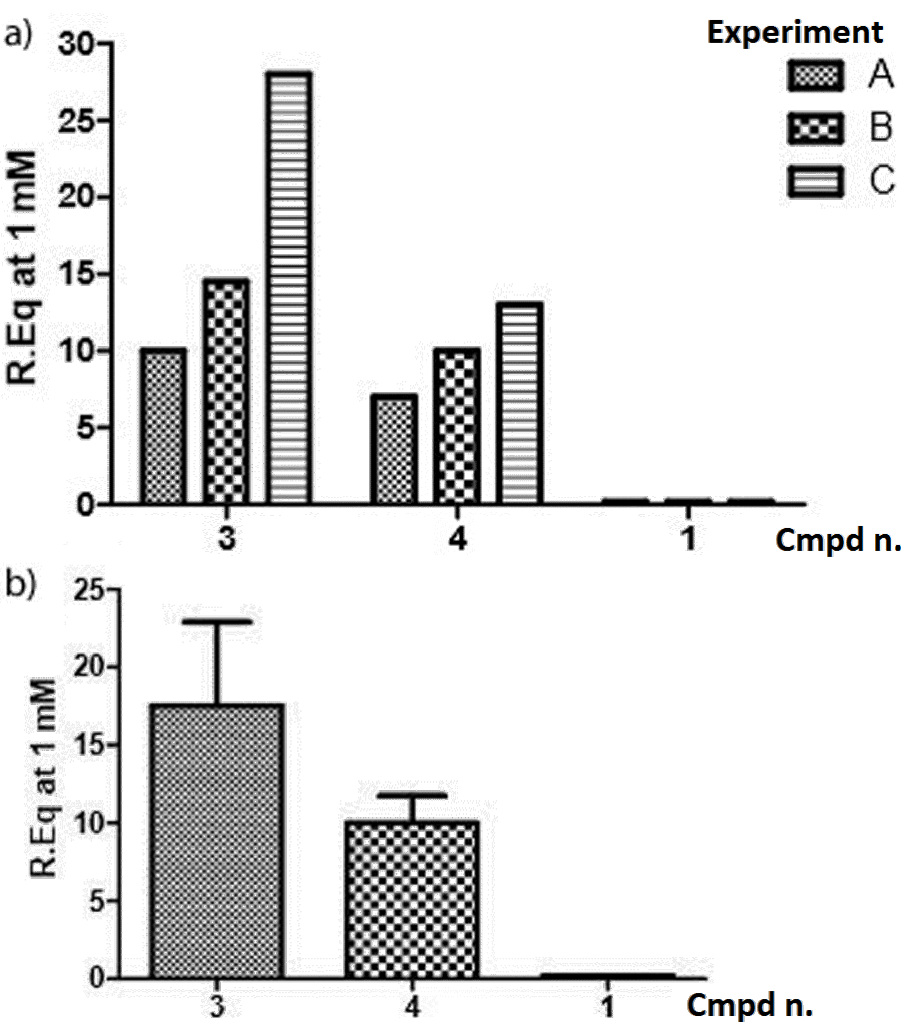
a) Relative affinities of glycocalixarene **1**, **3**, **4** (1 mM) towards Gal-3, expressed in terms of the increase of the resonance unit (RU) in three independent (A, B, C) SPR experiments. b) average affinities of glycocalixarenes with standard deviations.

The small increases of resonance units in the sensograms (Figure 3) showed a weak affinity of all calixarenes for Gal-3. However, the three synthetic molecules showed a very similar trend of Gal-3 binding affinity in three independent measurements (experiments A, B and C in Figure 4a). In particular, glycocalixarene **3** (cone structure, four lactosides) exhibited the highest affinity for Gal-3 in all experiments, while **4**, (1,3-alternate structure, four lactosides) displayed a lower affinity and **1** (cone structure, four galactosides) showed no interaction at all. The more efficient ligand, compound **3**, showed a dose-dependent affinity for the protein.

The higher affinities of lactose-containing compounds **3** and **4** for Gal-3 compared to galactose-containing compound **1** reflect the higher affinity of lactose over galactose for Gal-3. The lactosylcalixarene with the cone structure appears to bind better to Gal-3 than the corresponding isomer in the 1,3-alternate structure. This confirms the data previously obtained in a series of inhibition experiments of the same lectin in surface-immobilized asialofetuin and on cells with the lactosylthioureidocalixarenes (**I–III**) [20–21]. A direct comparison with ligands (**I–III**) was, however, not feasible since the lactosylthioreido derivatives tend to aggregate and precipitate under the conditions used for SPR experiments.

## Conclusion

Four glycocalixarenes **1–4** characterized by long hydrophilic spacers between the glycosyl units and the macrocyclic scaffold were synthesized by the copper(I)-catalyzed azido–alkyne cycloaddition (CuAAC). The homogeneous series of ligands **1**, **3** and **4** were subsequently studied in the binding to surface-immobilized His-tagged Gal-3 by SPR experiments. In spite of the weak intensity of the signals, an affinity order for the interaction of ligands **1**, **3** and **4** with the immobilized Gal-3 was obtained. A preference for the lactosyl clusters over the galactose functionalized ones (**3** > **4** >> **1**) and a higher efficiency in the binding of Gal-3 shown by the cone derivative compared to its isomeric 1,3-alternate counterpart (**3** > **4**) were observed. Work is in progress to study by SPR experiments the interaction between lactosylcalixarenes covalently immobilized on the chip and Gal-3 samples in solution.

## Supporting Information

Detailed experimental procedures (general information, synthetic procedures, expression and purification of Gal-3, Gal-3/glycocalixarene binding experiments), ^1^H NMR spectra of selected intermediates, and final glycocalixarenes **1–4** together with electrophoresis gel, circular dichroism spectrum and thermal unfolding of purified Gal-3.

File 1 Experimental part.

## References

[1] Lis H, Sharon N (1998). Chem Rev.

[2] Gabius H-J (1997). Eur J Biochem.

[3] Gabius H-J, Siebert H-C, André S, Jiménez-Barbero J, Rüdiger H (2004). ChemBioChem.

[4] Gabius H-J, André S, Jiménez-Barbero J, Romero A, Solis D (2011). Trends Biochem Sci.

[5] Ingrassia L, Camby I, Lefranc F, Mathieu V, Nshimyumukiza P, Darro F, Kiss R (2006). Curr Med Chem.

[6] Rubinstein N, Ilarregui J M, Toscano M A, Rabinovich G A (2004). Tissue Antigens.

[7] Sanchez-Ruderisch H, Fischer C, Detjen K M, Welzel M, Wimmel A, Manning J C, André S, Gabius H-J (2010). FEBS J.

[8] André S, Sanchez-Ruderisch H, Nakagawa H, Buchholz M, Kopitz J, Forberich P, Kemmner W, Böck C, Deguchi K, Detjen K M (2007). FEBS J.

[9] Baldini L, Casnati A, Sansone F, Ungaro R (2007). Chem Soc Rev.

[10] Dondoni A, Marra A (2010). Chem Rev.

[11] Sansone F, Rispoli G, Casnati A, Ungaro R, Renaudet O, Spinelli N (2011). Multivalent Glycocalixarenes. Synthesis and Biological Applications of Multivalent Glycoconjugates.

[12] Sansone F, Casnati A (2013). Chem Soc Rev.

[R13] 13Gutsche, C. D. *Calixarenes: An Introduction,* 2nd ed.; Royal Society of Chemistry: Cambridge, 2008. doi:10.1039/9781847558190

[14] Baldini L, Sansone F, Casnati A, Ungaro R, Steed J W, Gale P A (2012). Calixarenes in molecular recognition. Supramolecular Chemistry: from Molecules to Nanomaterials.

[15] Casnati A (1997). Gazz Chim Ital.

[16] Arosio D, Fontanella M, Baldini L, Mauri L, Bernardi A, Casnati A, Sansone F, Ungaro R (2005). J Am Chem Soc.

[17] Garcia-Hartjes J, Bernardi S, Weijers C A G M, Wennekes T, Gilbert M, Sansone F, Casnati A, Zuilhof H (2013). Org Biomol Chem.

[18] Cecioni S, Lalor R, Blanchard B, Praly J-P, Imberty A, Matthews S E, Vidal S (2009). Chem–Eur J.

[19] Moni L, Pourceau G, Zhang J, Meyer A, Vidal S, Souteyrand E, Dondoni A, Morvan F, Chevolot Y, Vasseur J-J (2009). ChemBioChem.

[20] André S, Grandjean C, Gautier F-M, Bernardi S, Sansone F, Gabius H-J, Ungaro R (2011). Chem Commun.

[21] André S, Sansone F, Kaltner H, Casnati A, Kopitz J, Gabius H-J, Ungaro R (2008). ChemBioChem.

[22] Dings R P M, Miller M C, Nesmelova I, Astorgues-Xerri L, Kumar N, Serova M, Chen X, Raymond E, Hoye T R, Mayo K H (2012). J Med Chem.

[23] Kolb H C, Finn M G, Sharpless K B (2001). Angew Chem, Int Ed.

[24] Renauldet O, Roy R (2014). Thematic issue: Multivalent scaffolds in glycosciences. Chem Soc Rev.

[25] Sansone F, Chierici E, Casnati A, Ungaro R (2003). Org Biomol Chem.

[26] Sansone F, Baldini L, Casnati A, Ungaro R (2008). Supramol Chem.

[27] Torvinen M, Neitola R, Sansone F, Baldini L, Ungaro R, Casnati A, Vainiotalo P, Kalenius E (2010). Org Biomol Chem.

[28] Consoli G M L, Cunsolo F, Geraci C, Mecca T, Neri P (2003). Tetrahedron Lett.

[29] Viola S, Consoli G M L, Merlo S, Drago F, Sortino M A, Geraci C (2008). J Neurochem.

[30] Consoli G M L, Cunsolo F, Geraci C, Sgarlata V (2004). Org Lett.

[31] Cardona F, Isoldi G, Sansone F, Casnati A, Goti A (2012). J Org Chem.

[32] Calvo-Flores F G, Isac-Garcia J, Hernandez-Mateo F, Pérez-Balderas F, Calvo-Asin J A, Sanchéz-Vaquero E, Santoyo-González F (2000). Org Lett.

[33] Dondoni A, Marra A (2006). J Org Chem.

[34] Rostovtsev V V, Green L G, Fokin V V, Sharpless K B (2002). Angew Chem, Int Ed.

[35] Tornoe C W, Christensen C, Meldal M (2002). J Org Chem.

[36] Bew S P, Brimage R A, L'Hermite N, Sharma S V (2007). Org Lett.

[37] Vecchi A, Melai B, Marra A, Chiappe C, Dondoni A (2008). J Org Chem.

[38] Cecioni S, Matthews S E, Blanchard H, Praly J-P, Imberty A, Vidal S (2012). Carbohydr Res.

[39] Aleandri S, Casnati A, Fantuzzi L, Mancini G, Rispoli G, Sansone F (2013). Org Biomol Chem.

[40] Bouillon C, Meyer A, Vidal S, Jochum A, Chevolot Y, Cloarec J-P, Praly J-P, Vasseur J-J, Morvan F (2006). J Org Chem.

[41] Sasaki A, Murahashi N, Yamada H, Morikawa A (1994). Biol Pharm Bull.

[42] Michel O, Ravoo B J (2008). Langmuir.

[43] Alyapyshev M Yu, Babain V A, Boyko V I, Eliseev I I, Kirsanov D O, Klimchuk O V, Legin A V, Mikhailina E S, Rodik R V, Smirnov I V (2010). J Inclusion Phenom Macrocyclic Chem.

[R44] 44No autocatalytic effect was observed even if the distance between the triazole unit and the macrocycle (3 atoms) is exactly the same.

[45] Kato H, Uzawa H, Nagatsuka T, Kondo S, Sato K, Ohsawa I, Kanamori-Kataoka M, Takei Y, Ota S, Furuno M (2011). Carbohydr Res.

[46] Xue J L, Cecioni S, He L, Vidal S, Praly J-P (2009). Carbohydr Res.

[47] Sansone F, Dudič M, Donofrio G, Rivetti C, Baldini L, Casnati A, Cellai S, Ungaro R (2006). J Am Chem Soc.

