# Supplementary material for: Clicked and long spaced galactosyl- and lactosylcalix[4]arenes: new multivalent galectin-3 ligands
Source: Beilstein J Org Chem. 2014 Jul 23;10:1672–80. doi: 10.3762/bjoc.10.175 (PMC4142870; doi:10.3762/bjoc.10.175)
Supplement: File 1 — Experimental part. [file Beilstein_J_Org_Chem-10-1672-s001.pdf]

Supporting Information

for

Clicked and long spaced galactosyl- and lactosylcalix[4]arenes: new multivalent galectin-3 ligands

Silvia Bernardi[1], Paola Fezzardi[1], Gabriele Rispoli[1], Stefania E. Sestito[2], Francesco Peri[*2], Francesco Sansone[1] and Alessandro Casnati[*1]

Address: [1]Dipartimento di Chimica, Università degli Studi di Parma, Parco Area delle Scienze 17/a, 43124 Parma, Italy and [2]Dipartimento di Biotecnologie e Bioscienze, Università degli Studi di Milano-Bicocca, Piazza della Scienza 2, 20126 Milano, Italy

Email: Francesco Peri – francesco.peri@unimib.it; Alessandro Casnati – casnati@unipr.it

*Corresponding author

Experimental part

Content:

- General information
- Synthetic procedures
- Expression and purification of Gal-3
- Gal-3/Glycocalixarene binding experiments

Figure S1: ^{1}H NMR spectrum (300 MHz, $CDCl_3$) of compound **5**.

Figure S2: ^{1}H NMR spectrum (400 MHz, $CDCl_3$) of compound **10**.

Figure S3: ^{1}H NMR spectrum (300 MHz, $CDCl_3$) of compound **15**.

Figure S4: ^{1}H NMR spectrum (300MHz, CD_3OD) of compound **12**.

Figure S5: ^{1}H NMR spectrum (400 MHz, CD_3OD) of compound **8**.

Figure S6: ^{1}H NMR spectrum (300 MHz, $CD_3OD/CDCl_3$ 9:1) of compound **18**.

Figure S7: ^{1}H NMR spectrum (400 MHz, $CD_3OD/CDCl_3$ 4:1) of compound **9**.

Figure S8: ^{1}H NMR spectrum (300 MHz, $CD_3OD/CDCl_3$ 3:1) of compound **13**.

Figure S9: ^{1}H NMR spectrum (300 MHz, CD_3OD) of compound **16**.

Figure S10: ^{1}H NMR spectrum (300 MHz, CD_3OD) of compound **19**.

Figure S11: ^{1}H NMR spectrum (300 MHz, CD_3OD) of compound **1**.

Figure S12: ^{1}H NMR spectrum (300 MHz, CD_3OD) of compound **2**.

Figure S13: ^{1}H NMR spectrum (300 MHz, CD_3OD) of compound **3**.

Figure S14: ^{1}H NMR spectrum (400 MHz, CD_3OD) of compound **4**.

Figure S15: electrophoresis gel of Gal-3 and circular dichroism spectrum and thermal unfolding of purified Gal-3.

General information

All moisture sensitive reactions were carried out under nitrogen atmosphere, using previously oven-dried glassware. All dry solvents were prepared according to standard procedures, distilled before use and stored over 3 Å or 4 Å molecular sieves. Most of the solvents and reagents were obtained from commercial sources and used without further purification. Analytical TLC was performed using prepared plates of silica gel (Merck 60 F_{254} on aluminum) and then, according to the functional groups present on the molecules, revealed with UV light or using staining reagents: $FeCl_3$ (1% in H_2O/CH_3OH 1:1), H_2SO_4 (5% in EtOH), ninhydrin (5% in EtOH), basic solution of $KMnO_4$ (0.75% in H_2O). Merck silica gel 60 (70–230 mesh) was used for flash chromatography and for preparative TLC plates. ^1H NMR and ^{13}C NMR spectra were recorded on Bruker AV300 and Bruker AV400 spectrometers (observation of ^1H nucleus at 300 MHz and 400 MHz respectively, and of ^{13}C nucleus at 75 MHz and 100 MHz respectively). All chemical shifts are reported in part per million (ppm) using the residual peak of the deuterated solvent, whose values are referred to tetramethylsilane (TMS, $\delta_{TMS} = 0$), as internal standard. All ^{13}C NMR spectra were performed with proton decoupling. Electrospray ionization (ESI) mass analyses were performed with a Waters spectrometer. HRMS-ESI spectra were recorded on a LTQ Orbitrap XL instrument. Gas chromatography mass analyses (GC–MS, electronic impact 70 eV) were recorded on a HP 6890 Series GC System apparatus, equipped with capillary column DB5 and quadrupolar mass selector HP 5973 Mass Selective Detector. Melting points were determined on an Electrothermal apparatus in closed capillaries. Microwave reactions were performed using a CEM Discovery System reactor.

2-(2-(2-Azidoethoxy)ethoxy)ethyl-2,3,4,6-tetra-*O*-acetyl-β-D-galactopyranoside (5)

Method 1) A solution of penta-*O*-acetyl-galactose (0.97 g, 2.50 mmol) in dry CH_2Cl_2 (5 mL) was cooled to 0 °C with an ice-water bath. At this temperature 2-(2-(2-azidoethoxy)ethoxy)ethanol (0.49 g, 2.80 mmol) and $BF_3 \cdot Et_2O$ (1.1 mL, 8.75 mmol) were added. The mixture was stirred at room temperature under N_2 for 24 h. The reaction was monitored via TLC (eluent: AcOEt/hexane 7:3). The reaction was then quenched by adding water (5 mL) and brine (2 mL) and then it was extracted with CH_2Cl_2 (3 x 10 mL). The combined organic phases were washed with water (20 mL) and brine (20 mL), dried over anhydrous Na_2SO_4, filtered and the solvent removed under reduced pressure. Attempts to purify the residue were made by using flash chromatography (elution

gradient: AcOEt/hexane 1:1 → 7:3) and preparative TLC plates (eluent: AcOEt/hexane 7:3), but it was not possible to obtain **5** as a pure compound. Yield: 79% (crude).

Method 2) NaN_3 (0.23 g, 3.60 mmol) and tetrabutylammonium iodide (0.53 g, 1.44 mmol) were added to a solution of penta-*O*-acetyl-β-galactose (0.36 g, 0.72 mmol) in dry DMF (5 mL). The mixture was stirred at 90 °C for 48 hours under nitrogen atmosphere. ESIMS analyses were performed to monitor the reaction. The solvent was subsequently removed in vacuo and the residue redissolved in AcOEt (30 mL). The organic phase was washed with water (2 × 30 mL), dried over anhydrous Na_2SO_4, filtered and the solvent removed under reduced pressure. Purification via column chromatography (eluent: AcOEt/petroleum ether 1:1) gave pure product **5** as a light yellow oil. Yield: 72%.

^{1}H NMR (300 MHz, $CDCl_3$): δ (ppm) 5.37 (dd, 1H, $J_{4\text{-}5}$ = 1.2 Hz, $J_{4\text{-}3}$ = 4.0 Hz, H_4); 5.19 (dd, 1H, $J_{1\text{-}2}$ = 8.1 Hz, $J_{2\text{-}3}$ = 10.5 Hz, H_2); 5.00 (dd, 1H, $J_{3\text{-}4}$ = 4.0 Hz, $J_{2\text{-}3}$ = 10.5 Hz, H_3); 4.56 (d, 1H, $J_{1,2}$ = 8.1 Hz, H_1); 4.19-4.08 (m, 2H, H_{6a}, H_{6b}); 3.98-3.87 (m, 2H, β-OCH_a, H_5); 3.81-3.71 (m, 1H, β-OCH_b); 3.70-3.59 (m, 8H, CH_2 ethylene glycol chain); 3.87 (t, 2H, J = 5.1 Hz, CH_2N_3); 2.14, 2.05, 2.04, 1.97 (4s, 12H, Ac). ESI-MS(+) m/z: 528.2 [100, $(M+Na)^+$]. The product shows the same physical and spectroscopic properties reported in the literature [1].

2-(2-(2-Propargyloxyethoxy)ethoxy)ethyl-2,3,4,6-tetra-*O*-acetyl-β-D-galactopyranoside (10)

A solution of propargyl functionalized triethylene glycol (0.68 g, 3.60 mmol) in 5 mL of dry CH_2Cl_2 was added to a solution of penta-*O*-acetyl-β-galactose (1.40 g, 3.60 mmol) in 10 mL of dry CH_2Cl_2. The mixture was cooled to 0 °C and then $BF_3{\cdot}Et_2O$ (2.28 mL, 18.0 mmol) was slowly added. The reaction was stirred at 0 °C for 1 h and then allowed to slowly reach room temperature and further stirred for 14 h under nitrogen atmosphere. The reaction was monitored via TLC (eluent: CH_2Cl_2/AcOEt 7:3). The mixture was then poured into a $NaHCO_3$ aqueous saturated solution (20 mL) and it was stirred till the formation of gas was not observed any longer. The aqueous phase was extracted with CH_2Cl_2 (3 × 20 mL). The combined organic phases were washed with water (20 mL), dried over anhydrous Na_2SO_4, filtered and the solvent removed under reduced pressure. The crude was purified by flash chromatography, first with an elution gradient: CH_2Cl_2/AcOEt 9:1 → 7:3 and then with petroleum ether/AcOEt 1:1. Product **10** was obtained pure as a yellow oil. Yield: 43%. ^{1}H NMR (400 MHz, $CDCl_3$): δ (ppm) 5.34 (d, 1H, J = 3.2 Hz, H_4); 5.16 (dd, 1H, $J_{1\text{-}2}$ = 8.0 Hz, $J_{2\text{-}3}$ = 10.4 Hz, H_2); 4.97 (dd, 1H, $J_{3\text{-}4}$= 3.2 Hz, $J_{2\text{-}3}$ = 10.4 Hz, H_3); 4.54 (d, 1H, J = 8.0 Hz, H_1); 4.16 (d, 2H, J = 2.4 Hz, CH_2CCH); 4.14–4.07 (m, 2H, H_{6a}, H_{6b}); 3.92–3.86 (m, 2H, H-5, β-OCH_a); 3.74–3.60 (m, 11H, β-OCH_b, ethylene glycol chain); 2.41 (t, 2H, J = 2.4 Hz, C≡C*H*); 2.11, 2.02, 2.01, 1.94 (4s, 12H,

$COCH_3$). ESI-MS(+) m/z: 541.8 [100%, $(M+Na)^+$]. The product shows the same physical and spectroscopic properties reported in the literature [2].

***Cone*-5,11,17,23-tetrakis[pent-4-ynoylamino]-25,26,27,28-tetrapropoxy-calix[4]arene (8)**

Method 1) 4-Pentynoic acid (0.18 g, 1.84 mmol) and DCC (0.38 g, 1.84 mmol) were dissolved in 3 mL CH_2Cl_2. The solution was stirred for 10 min and then amino-calix[4]arene **6** [3] (0.20 g, 0.31 mmol) and a catalytic amount of DMAP were added. The reaction was refluxed for 5 hours, checking the progress via TLC (CH_2Cl_2/CH_3OH 9:1). The formed precipitate was then filtered off and the filters were washed with CH_2Cl_2 and CH_3OH. The organic phase was collected and the solvent removed in vacuum. The residue was purified by flash chromatography (eluent: CH_2Cl_2/CH_3OH 97:3) and preparative TLC plates (eluent: CH_2Cl_2/CH_3OH 95:5) giving compound **8** as a white solid. Yield: 44%.

Method 2) 4-Pentynoic acid (0.18 g, 1.84 mmol) and EDC (0.35 mg, 1.84 mmol) were dissolved in 14 mL CH_2Cl_2 and 6 mL pyridine. The solution was stirred for 30 min at room temperature and then amino-calix[4]arene **6** [3] (0.20 g, 0.31 mmol) were added. The mixture turned immediately red. The reaction was stirred at room temperature for 18 hours and it was monitored via TLC (eluent: CH_2Cl_2/CH_3OH 9:1). The mixture was then diluted with CH_2Cl_2 (20 mL) and washed with 1M HCl (2 × 20 mL) and water (20 mL). The combined aqueous phases were extracted with CH_2Cl_2 (30 mL). The organic layers were collected together and evaporated to dryness. The residue was purified via column chromatography (eluent: CH_2Cl_2/CH_3OH 97:3) to give product **8** as a white solid. Yield: 66%

^{1}H NMR (300 MHz, CD_3OD): δ (ppm) 6.88 (s, 8H, Ar); 4.46 (d, 4H, J = 13.2 Hz, *ax*-$ArCH_2Ar$); 3.85 (t, 8H, J = 7.5 Hz, $OCH_2CH_2CH_3$); 3.11 (d, 4H, J = 13.2 Hz, *eq*-$ArCH_2Ar$); 2.49–2.46 (m, 16H, $COCH_2CH_2$, $COCH_2CH_2$); 2.27 (bs, 4H, C≡CH), 2.00–1.93 (m, 8H, $OCH_2CH_2CH_3$); 1.03 (t, 12H, J = 7.2 Hz, $OCH_2CH_2CH_3$). ^{13}C NMR (100 MHz, CD_3OD): δ (ppm) 170.4 (CO); 153.1 (Ar ipso); 134.8 (Ar ortho); 132.1 (Ar para); 120.5 (Ar meta); 82.1 (*C*≡CH); 76.7 ($OCH_2CH_2CH_3$); 69.0 (C≡*C*H); 35.3 ($COCH_2CH_2$); 30.7 ($ArCH_2Ar$); 22.9 ($OCH_2CH_2CH_3$); 14.1 ($COCH_2CH_2$); 9.4 ($OCH_2CH_2CH_3$). HRMS (ESI) m/z: $[M + Na]^+$ Calcd for $C_{60}H_{68}N_4O_8Na$ 995.49293; Found 995.49312.

***Cone*-5,11,17,23-tetrakis[α-chloroacetamido]-25,26,27,28-tetrapropoxy-calix[4]arene (11)**

N,*N*-Diisopropylethylamine (0.53 mL, 3.06 mmol) and chloroacetyl chloride (0.24 mL, 3.06 mmol) were added to a solution of amino-calix[4]arene **6** (200 mg, 0.306 mmol) in dry CH_2Cl_2 (10 mL). The mixture was stirred at room temperature for 6 h under nitrogen atmosphere. The progress of

the reaction was monitored via TLC (eluent: AcOEt/hexane 7:3). The reaction was quenched by stirring the mixture for 10 min with 0.5 M HCl (10 mL). The aqueous phase was then extracted with CH_2Cl_2 (2 × 20 mL). The combined organic phases were washed with water till neutral pH, dried over anhydrous Na_2SO_4, filtered and the solvent removed under reduced pressure. The residue was recrystallized from Et_2O/AcOEt 95:5. Product **11** was obtained as a light brown solid. M.p. : 208-210 °C. Yield: 55**%.** ^{1}H NMR (300 MHz, $CDCl_3$/CD_3OD 9:1): δ (ppm) 6.75 (s, 8H, Ar); 4.31 (d, 4H, J = 13.2 Hz, *ax*-ArCH_2Ar); 3.94 (s, 8H, CH_2Cl); 3.70 (t, 8H, J = 7.4 Hz, O$CH_2CH_2CH_3$); 3.01 (d, 4H, J = 13.2 Hz, *eq*-ArCH_2Ar); 1.88-1.73 (m, 8H, O$CH_2CH_2CH_3$); 0.87 (t, 12H, J = 7.4 Hz, O$CH_2CH_2CH_3$). ESI-MS (+)m/z: 981 [100%, $(M+Na)^+$]. The spectroscopic data found are in agreement with those reported in literature [4].

***Cone*-5,11,17,23-tetrakis[α-azidoacetamido]-25,26,27,28-tetrapropoxy-calix[4]arene (12)**

Calix[4]arene **11** (290 mg, 0.30 mmol) was dissolved in 3 mL DMF and diluted to 30 mL with CH_3OH. NaN_3 (312 mg, 4.80 mmol) was then added and the mixture was refluxed for 1 hour. The reaction was monitored via TLC (eluent: hexane/THF 6:4) and ESIMS analyses. When complete, the solvent was removed under reduced pressure. The residue was redissolved in CH_2Cl_2 and filtered to remove the inorganic salts. The organic phase was collected, brought to dryness and the crude purified via recrystallization from hexane. Product **12** was obtained as a yellow solid. M.p. : 201-203 °C. Yield: 75%. ^{1}H NMR (300 MHz, CD_3OD): δ (ppm) 6.95 (s, 8H, ArH); 4.50 (d, 4H, J = 13.2 Hz, *ax*-ArCH_2Ar); 3.95-3.85 (m, 16H, O$CH_2CH_2CH_3$, CH_2N_3); 3.16 (d, 4H, J = 13.2 Hz, *eq*-ArCH_2Ar); 2.06-1.90 (m, 8H, O$CH_2CH_2CH_3$); 1.04 (t, 12H, J = 7.4 Hz, O$CH_2CH_2CH_3$). ^{13}C NMR (300 MHz, CD_3OD): δ (ppm) 158.8 (CONH); 145.2 (Ar ipso); 136.6 (Ar ortho); 133.4 (Ar para); 122.1 (Ar meta); 78.5 (O$CH_2CH_2CH_3$); 53.4 (CH_2N_3); 32.4 (ArCH_2Ar); 24.7 (O$CH_2CH_2CH_3$); 11.1 (O$CH_2CH_2CH_3$). HRMS (ESI) m/z: $[M + Na]^+$ Calcd for $C_{48}H_{56}N_{16}O_8Na$ 1007.43592; Found 1007.43656.

2-(2-(2-Chloroethoxy)ethoxy)ethyl-2,3,4,6-tetra-*O*-acetyl-β-D-galactopyranosyl-(1→4)-2,3,6-tri-*O*-acetyl-β-D-glucopyranoside (14)

Method 1) Octa-*O*-acetyl-lactose (2.03 g, 3.00 mmol) and 2-(2-(2-chloroethoxy)ethoxy) ethanol (0.65 mL, 4.50 mmol) were dissolved in 10 mL dry CH_2Cl_2. The mixture was cooled to 0°C with an ice-water bath and $BF_3{\cdot}Et_2O$ (1.90 mL, 15.0 mmol) was slowly added. The mixture, kept under N_2, was allowed to slowly reach room temperature and it was stirred for 24 h. The reaction was checked via TLC (eluent: AcOEt/petroleum ether 7:3). Subsequently, $NaHCO_3$ aqueous saturated solution (30 mL) was added for quenching. The aqueous phase was extracted with CH_2Cl_2 (3 × 30

mL). The combined organic phases were washed with water (30 mL), dried over anhydrous Na_2SO_4, filtered and the solvent removed under reduced pressure. The residue was purified by flash chromatography (elution gradient: AcOEt/petroleum ether 65:35 → 8:2). Product **14** (light yellow oil) was obtained as a mixture of α and β anomers. Yield: 26%.

Method 2) Octa-*O*-acetyl-lactose (2.03 g, 3.00 mmol) and 2-(2-(2-chloroethoxy)ethoxy) ethanol (0.65 mL, 4.50 mmol) were dissolved in 10 mL dry CH_2Cl_2. CF_3COOAg (0.99 g, 4.50 mmol) and a 1 M solution of $SnCl_4$ (0.91 mL, 9.00 mmol) in dry CH_2Cl_2 was added dropwise under argon atmosphere. The reaction was stirred at room temperature under Ar for 3 hours. The reaction was monitored via TLC (AcOEt/petroleum ether 7:3). $NaHCO_3$ saturated aqueous solution (30 mL) was subsequently added for quenching. The precipitate was filtered off through a Celite pad. The organic phase was collected and the aqueous phase was extracted with CH_2Cl_2 (4 × 30 mL). The combined organic phases were washed with water and brine till neutral pH, dried over anhydrous Na_2SO_4, filtered and the solvent removed under reduced pressure. The residue was purified by flash chromatography (elution gradient: AcOEt/hexane 65:35 →7:3). Product **14** was obtained pure as a yellow oil. Yield: 74%.

^{1}H NMR (300 MHz, $CDCl_3$): δ(ppm) 5.32 (d, 1H, J = 3.3 Hz, H_4'); 5.17 (t, 1H, J = 9.3 Hz, H_3); 5.08 (dd, 1H, $J_{1'-2'}$ = 7.9 Hz, $J_{2'-3'}$ = 10.4 Hz, H_2'); 4.93 (dd, 1H, $J_{2'-3'}$ = 10.4 Hz, $J_{3'-4'}$ = 3.3Hz, H_3'); 4.87 (dd, 1H, J_{1-2} = 7.8 Hz, J_{2-3} = 9.3 Hz, H_2); 4.55 (d, 1H, J = 7.8 Hz, H_1); 4.51-4.38 (m, 2H, H_1', H_{6a}); 4.20-4.00 (m, 3H, H_{6b}, $H_6'_a$, $H_6'_b$); 3.93-3.79 (m, 2H, H_5', β-OCH_a); 3.78-3.68 (m, 4H, H_4, β-OCH_b, CH_2 ethylene glycol chain); 3.67-3.53 (m, 9H, H_5, 3CH_2 ethylene glycol chain, CH_2Cl), 2.13, 2.12, 2.10, 2.09, 2.04, 2.02, 1.94 (7s, 21H, $COCH_3$). ^{13}C NMR (300 MHz, $CDCl_3$): δ(ppm) 170.3, 170.1, 170.0, 169.7, 169.6, 169.5, 169.0 (CH_3C=O); 101.1 (C_1'); 100.6 (C_1); 76.3 (C_4); 72.8 (C_3); 72.6 (C_5); 71.7 (C_2); 71.4 (CH_2 ethylene glycol chain); 71.0 (C_3'), 70.7, 70.6 (C_5', 2CH_2 ethylene glycol chain); 70.3,70.1 (2CH_2 ethylene glycol chain); 69.1 (C_2', β-OCH_2); 66.6 (C_4'); 62.0 (C_6); 60.8 (C_6'); 42.8 (CH_2Cl); 20.9, 20.8, 20.7, 20.6, 20.5 (CH_3C=O). ESI-MS(+) m/z: 809.2 [100%, $(M+Na)^+$]. The product shows the same physical and spectroscopic characteristics reported in literature [5].

2-(2-(2-Azidoethoxy)ethoxy)ethyl-2,3,4,6-tetra-*O*-acetyl-β-D-galactopyranosyl-(1→4)-2,3,6-tri-*O*-acetyl-β-D-glucopyranoside (15)

NaN_3 (0.15 g, 2.35 mmol) and tetrabutylammonium iodide (0.35 g, 0.94 mmol) were added to a solution of lactoside derivative **14** (0.37 g, 0.47 mmol) in dry DMF (10 mL). The mixture was stirred at 90 °C for 24 hours under nitrogen atmosphere. ESIMS analyses were performed to monitor the reaction. The solvent was subsequently removed in vacuo and the residue redissolved in AcOEt (40

mL). The organic phase was washed with water (2 × 30 mL), dried over anhydrous Na_2SO_4, filtered and the solvent removed under reduced pressure. Purification via column chromatography (elution gradient: AcOEt/toluene 65:35 → 7:3) gave pure product **15** as a light yellow oil. Yield: 60%. ^{1}H NMR (300 MHz, $CDCl_3$): δ (ppm) 5.25 (d, 1H, J = 3.3 Hz, H_4'); 5.10 (t, 1H, J = 9.3Hz, H_3); 5.01 (dd, 1H, $J_{1'-2'}$ = 7.9 Hz, $J_{2',3'}$ = 10.4 Hz, H_2'); 4.87 (dd, 1H, $J_{2'-3'}$ = 10.4 Hz, $J_{3'-4'}$ = 3.3 Hz, H_3'); 4.87 (dd, 1H, J_{1-2} = 7.9 Hz, J_{2-3} = 9.3 Hz, H_2); 4.49 (d, 1H, J = 7.9 Hz, H_1); 4.44-4.36 (m, 2H, H_1', H_{6a}); 4.09-3.94 (m, 3H, H_{6b}, H_6'$_a$, H_6'$_b$); 3.88-3.76 (m, 2H, H_5', β-OCH_a); 3.71 (t, 1H, J = 9.3 Hz, H_4); 3.67-3.47 (m, 10H, H_5, β-OCH_b, CH_2 ethylene glycol chain); 3.31 (t, 2H, J = 5.0 Hz, CH_2N_3); 2.06 (s, 3H, $COCH_3$); 2.03 (s, 3H, $COCH_3$); 2.00-1.92 (m, 12H, $COCH_3$); 1.88 (s, 3H, $COCH_3$). ^{13}C NMR (300 MHz, $CDCl_3$): δ (ppm) 170.3, 170.2, 170.1 , 170.0, 169.7, 169.6, 169.0 (CH_3*C*O); 101.0 (C_1'); 100.5 (C_1); 76.2 (C_4); 72.8 (C_3); 72.5 (C_5); 71.6 (C_2); 70.9 (C_3'); 70.7, 70.6 (C_5', 2CH_2 ethylene glycol chain); 70.3,70.0 (2CH_2 ethylene glycol chain); 69.0 (C_2', β-OCH_2); 66.6 (C_4'); 62.0 (C_6); 60.8 (C_6'); 50.6 (CH_2N_3); 20.8, 20.7, 20.6, 20.5, 20.4 (*C*H_3CO). ESI-MS(+) m/z: 816.2 [100%, $(M+Na)^+$]. The product shows the same physical and spectroscopic properties reported in the literature [5].

***Alt*-5,11,17,23-tetrakis[pent-4-ynoylamino]-25,26,27,28-tetrapropoxy-calix[4]arene (18)**

4-Pentynoic acid (90.0 mg, 0.92 mmol) and EDC (176.4 mg, 0.92 mmol) were dissolved in 7 mL CH_2Cl_2 and 3 mL pyridine. The solution was stirred for 30 min at room temperature and then *alt*-tetra-amino calix[4]arene **17** [6,7] (98.1 mg, 0.15 mmol) was added. The mixture turned immediately red. The reaction was stirred at room temperature for 18 hours and it was monitored via TLC (eluent: CH_2Cl_2/CH_3OH 9:1). The mixture was then diluted with CH_2Cl_2 (10 mL) and washed with 1 M HCl (2 × 10 mL) and water (2 × 10 mL). The combined aqueous phases were extracted with CH_2Cl_2 (30 mL). The organic layers were collected together and evaporated to dryness. The residue was purified via column chromatography (eluent: CH_2Cl_2/CH_3OH 97:3) to give product **18** as a white solid. Yield: 65%. ^{1}H NMR (300 MHz, $CD_3OD/CDCl_3$ 9:1): δ (ppm) 7.31 (s, 8H, Ar); 3.73 (s, 8H, $ArCH_2Ar$); 3.24 (t, 8H, J = 7.4 Hz, $OCH_2CH_2CH_3$); 2.57-2.45 (m, 16H, $COCH_2CH_2$, $COCH_2CH_2$); 2.18-2.14 (m, 4H, C≡CH), 1.34-1.23 (m, 8H, $OCH_2CH_2CH_3$); 0.67 (t, 12H, J = 7.4 Hz, $OCH_2CH_2CH_3$). ^{13}C NMR (100 MHz, $CD_3OD/CDCl_3$ 9:1): δ (ppm) 171.8 (CO); 154.9 (Ar ipso); 135.7 (Ar ortho); 134.1 (Ar para); 122.5 (Ar meta); 83.8 (*C*≡CH); 73.9 ($OCH_2CH_2CH_3$); 70.5 (C≡*C*H); 39.6 ($ArCH_2Ar$); 37.2 ($COCH_2CH_2$); 24.1 ($OCH_2CH_2CH_3$); 16.2 ($COCH_2CH_2$); 10.9 ($OCH_2CH_2CH_3$). HRMS (ESI) m/z: $[M + Na]^+$ Calcd for $C_{60}H_{68}N_4O_8Na$ 995.49293; Found 995.49329.

General procedure for "click" reactions (compounds 9, 13, 16, 19)

Calix[4]arene derivative (1 equiv) and β-galactoside derivative (6 equiv) were dissolved in 2.5 mL of DMF in a microwave tube. $CuSO_4 \cdot 5H_2O$ (0.3 or 0.6 equiv), sodium ascorbate (0.6 or 1.2 equiv) and 0.5 mL H_2O were then added. The mixture was heated at 80 °C by microwave irradiation (150 W) for a reaction time between 20 and 40 minutes. When the reaction was completed (checked via TLC and ESIMS), it was quenched by addition of water (15 mL) and extracted with AcOEt (5 x 15 mL). The combined organic layers were dried over anhydrous Na_2SO_4, filtered and the solvent removed under reduced pressure. The crude was purified by flash chromatography to afford pure the peracetylated glycosyl calix[4]arene.

***Cone*-peracetylated-galactosyl-calix[4]arene (9)**

Cone-tetra-alkyne calix[4]arene **8** (38.9 mg, 0.04 mmol), azido galactoside **5** (121.3 mg, 0.24 mmol), $CuSO_4 \cdot 5H_2O$ (3.0 mg 12.0 µmol), sodium ascorbate (4.7 mg, 24.0 µmol). Reaction time: 20 min. TLC (eluent: CH_2Cl_2/CH_3OH 95:5). Flash chromatography (elution gradient: CH_2Cl_2/CH_3OH 95:5 → 93:7). Product **9** was obtained as a light yellow solid. Yield: 83%. ^1H NMR (400 MHz, $CD_3OD/CDCl_3$ 4:1): δ(ppm) 7.75 (bs, 4H, C*H* triazole); 6.83 (s, 8H, Ar); 5.35 (s, 4H, H_4); 5.09-5.07 (m, 8H, H_2, H_3); 4.64 (d, 4H, J = 6.8 Hz, H_1); 4.52 (bs, 8H, NCH_2CH_2); 4.41 (d, 4H, J = 13.2 Hz, *ax*-$ArCH_2Ar$); 4.12-4.10 (m, 8H, H_{6a}, H_{6b}); 4.05-4.02 (m, 4H, H_5); 3.93-3.90 (m, 4H, β-OCH_a); 3.84-3.79 (m, 16H, NCH_2CH_2, $OCH_2CH_2CH_3$); 3.70-3.67 (m, 4H, β-OCH_b); 3.58-3.53 (m, 24H, ethylene glycol chain); 3.09 (d, 4H, J = 13.2 Hz, *eq*-$ArCH_2Ar$); 3.02 (bs, 8H, $COCH_2CH_2$triazole); 2.62 (bs, 8H, $COCH_2CH_2$triazole); 2.10, 2.01, 1.99 (3s, 36H, $COCH_3$); 1.93 (s, 20H, $COCH_3$, $OCH_2CH_2CH_3$); 0.98 (t, 12H, J = 7.2 Hz, $OCH_2CH_2CH_3$). ^{13}C NMR (100 MHz, $CD_3OD/CDCl_3$ 4:1): δ(ppm) 170.7, 170.6, 170.2, 170.0 (CH_3CO); 153.1 (Ar ipso); 146.3 (C_q triazole); 134.9 (Ar ortho); 132.1 (Ar para); 123.3 (CH triazole); 120.6 (Ar meta); 100.9 (C_1); 76.8 ($OCH_2CH_2CH_3$); 71.0, 69.0 (C_2, C_3); 70.4 (C_5); 70.3, 70.2, 70.0 (CH_2 ethylene glycol chain); 69.0 (NCH_2CH_2, β-OCH_2); 67.4 (C_4); 61.2 (C_6); 50.2 (NCH_2); 35.8 ($COCH_2CH_2$triazole); 30.9 ($ArCH_2Ar$); 23.0 ($OCH_2CH_2CH_3$); 21.2 ($COCH_2CH_2$triazole); 19.9, 19.7 ($COCH_3$); 9.7 ($OCH_2CH_2CH_3$). HRMS (ESI) m/z: $[M + 2Na]^{2+}$ Calcd for $C_{140}H_{192}N_{16}O_{56}Na_2$ 1519.62262; Found 1519.62348.

***Cone*-peracetylated-galactosyl-calix[4]arene (13)**

Cone-tetra-azido calix[4]arene **12** (39.4 mg, 0.04 mmol), alkyne galactoside **10** (124.4 mg, 0.24 mmol), $CuSO_4 \cdot 5H_2O$ (3.0 mg 12.0 µmol), sodium ascorbate (4.7 mg, 24.0 µmol). Reaction time: 20 min. TLC (eluent: CH_2Cl_2/CH_3OH 95:5). Flash chromatography (elution gradient: CH_2Cl_2/CH_3OH 95:5

→ 93:7). Product **13** was obtained as a light yellow solid. Yield: 81%. ^{1}H NMR (300 MHz, $CD_3OD/CDCl_3$ 3:1): δ (ppm) 7.89 (bs, 4H, CH triazole); 6.98 (bs, 8H, Ar); 5.35 (d, 4H, J = 2.1 Hz, H_4); 5.13-5.08 (m, 16H, H_2, H_3, $COCH_2$triazole); 4.65 (d, 4H, J = 7.2 Hz, H_1); 4.58 (bs, 8H, OCH_2triazole); 4.46 (d, 4H, J = 12.9 Hz, *ax*-$ArCH_2Ar$); 4.13-4.10 (m, 8H, H_{6a}, H_{6b}); 4.05-4.00 (m, 4H, H_5); 3.93-3.85 (m, 12H, β-OCH_a, $OCH_2CH_2CH_3$); 3.74-3.59 (m, 44H, β-OCH_b, ethylene glycol chain); 3.12 (d, 4H, J = 12.9 Hz, *eq*-$ArCH_2Ar$); 2.12 (s, 12H, CH_3CO); 2.03-1.92 (m, 44H, CH_3CO, $OCH_2CH_2CH_3$); 1.00 (t, 12H, J = 7.2 Hz, $OCH_2CH_2CH_3$). ^{13}C NMR (75 MHz, $CD_3OD/CDCl_3$ 3:1): 170.7, 170.6, 170.2, 170.1 (CH_3CO); 163.6 (CONH); 153.3 (Ar ipso); 144.8 (C_q triazole); 135.1 (Ar ortho); 131.9 (Ar para); 125.6 (CH triazole); 120.1 (Ar meta); 100.9 (C_1); 77.0 ($OCH_2CH_2CH_3$); 71.0, 69.0 (C_2, C_3); 70.4 (C_5); 70.3, 70.2, 70.1 (ethylene glycol chain); 69.5 (β-OCH_2CH_2); 68.9 (β-OCH_2); 67.4 (C_4); 63.7 (OCH_2triazole); 61.3 (C_6); 52.4 ($COCH_2$triazole); 31.0 ($ArCH_2Ar$); 23.0 ($OCH_2CH_2CH_3$); 20.0, 19.8, 19.7 ($COCH_3$); 9.7 ($OCH_2CH_2CH_3$). HRMS (ESI) m/z: $[M + 3Na]^{3+}$ Calcd for $C_{140}H_{192}N_{16}O_{60}Na_3$ 1042.07137; Found 1042.07182.

Cone-peracetylated-lactosyl-calix[4]arene (16)

Cone-tetra-alkyne calix[4]arene **8** (23.5 mg, 24.1 μmol), azido lactoside **15** (119.8 mg, 0.15 mmol), $CuSO_4·5H_2O$ (3.6 mg 14.5 μmol), sodium ascorbate (5.7 mg, 28.9 μmol). Reaction time: 40 min. TLC (eluent: CH_2Cl_2/CH_3OH 95:5). Flash chromatography (elution gradient: CH_2Cl_2/CH_3OH 97:3 → 94:6). Product **16** was obtained as a yellow oil. Yield: 46%.

^{1}H NMR (300 MHz, CD_3OD): δ (ppm) 7.78 (bs, 4H, CH triazole); 6.89 (bs, 4H, Ar); 6.87 (bs, 4H, Ar); 5.33 (d, 4H, J = 3.3 Hz, H_4'); 5.16 (t, 4H, J = 9.2 Hz, H_3); 5.10 (dd, 4H, $J_{2'-3'}$ = 10.4 Hz, $J_{3'-4'}$ = 3.3 Hz, H_3'); 4.99 (dd, 4H, $J_{1'-2'}$ = 7.8 Hz, $J_{2',3'}$ = 10.4 Hz, H_2'); 4.82 (dd, 4H, J_{1-2} = 7.8 Hz, J_{2-3} = 9.2 Hz, H_2); 4.73-4.63 (m, 8H, H_1, H_1'); 4.62-4.40 (m, 16H, H_{6a}, *ax*-$ArCH_2Ar$, NCH_2); 4.21-4.06 (m, 16H, H_5', H_{6b}, $H_6'_a$, $H_6'_b$); 3.94-3.78 (m, 24H, H_4, β-OCH_a, NCH_2CH_2, $OCH_2CH_2CH_3$); 3.77-3.61 (m, 8H, H_5, β-OCH_b); 3.60-3.43 (m, 24H, CH_2 ethylene glycol chain); 3.12 (d, 4H, J = 13.0 Hz, *eq*-$ArCH_2Ar$); 3.01 (t, 8H, J = 6.8 Hz, $COCH_2CH_2$triazole); 2.63 (t, 8H, J = 6.8 Hz, $COCH_2CH_2$triazole); 2.14, 2.11 (2s, 24H, $COCH_3$); 2.06-1.87 (m, 68H, $COCH_3$, $OCH_2CH_2CH_3$); 1.02 (t, 12H, J = 7.4, $OCH_2CH_2CH_3$). ^{13}C NMR (75 MHz, CD_3OD): δ (ppm) 170.9, 170.7, 170.6, 170.5, 170.3, 170.0, 169.9, 169.7 (CH_3CO, CONH); 153.0 (Ar ipso); 146.2 (C_q triazole); 134.8 (Ar ortho); 132.3 (Ar para); 122.9 (CH triazole); 120.4 (Ar meta); 100.6, 100.4 (C_1, C_1'); 76.8 ($OCH_2CH_2CH_3$); 76.3 (C_4); 73.1 (C_3); 72.6 (C_5); 71.7 (C_2); 71.1 (C_3'); 70.4 (C_5); 70.3, 70.1, 70.0, 69.9, 69.3, 69.1, 68.9 (C_5', C_2', β-OCH_2, 3CH_2 ethylene glycol chain, NCH_2CH_2,); 67.2 (C_4'); 62.3 (C_6); 60.8 (C_6'); 50.0 (NCH_2); 35.6 ($COCH_2CH_2$triazole); 30.7 ($ArCH_2Ar$); 23.0 ($OCH_2CH_2CH_3$); 20.8 ($COCH_2CH_2$triazole); 19.8, 19.5, 19.4, 19.3, 19.1 ($COCH_3$); 9.4

($OCH_2CH_2CH_3$). HRMS (ESI) m/z: $[M + 3Na]^{3+}$ Calcd for $C_{188}H_{256}N_{16}O_{88}Na_3$ 1404.85751; Found 1404.85826.

Alt-peracetylated-lactosyl-calix[4]arene (19)

Alt-tetra-alkyne calix[4]arene **18** (11.9 mg, 12.2 µmol), azido lactoside **15** (58.1 mg, 73.2 µmol), $CuSO_4\cdot5H_2O$ (1.8 mg 7.3 µmol), sodium ascorbate (2.9 mg, 14.6 µmol). Reaction time: 40 min. TLC (eluent: CH_2Cl_2/CH_3OH 95:5). Flash chromatography (elution gradient: CH_2Cl_2/CH_3OH 97:3 → 94:6). Product **19** was obtained as a yellow oil. Yield: 46%. ^1H NMR (300 MHz, CD_3OD): δ (ppm) 7.80 (bs, 4H, CH triazole); 7.33 (bs, 4H, Ar); 7.32 (bs, 4H, Ar); 5.33 (d, 4H, J = 3.3 Hz, H_4'); 5.16 (t, 4H, J = 9.2 Hz, H_3); 5.10 (dd, 4H, $J_{2'-3'}$ = 10.4 Hz, $J_{3'-4'}$ = 3.3 Hz, H_3'); 4.99 (dd, 4H, $J_{1'-2'}$ = 7.8 Hz, $J_{2',3'}$ = 10.4 Hz, H_2'); 4.82 (dd, 4H, J_{1-2} = 7.8 Hz, J_{2-3}= 9.2 Hz, H_2); 4.73-4.63 (m, 8H, H_1, H_1'); 4.58-4.43 (m, 12H, H_{6a}, NCH_2); 4.19-4.05 (m, 16H, H_5', H_{6b}, $H_6'_a$, $H_6'_b$); 3.94-3.80 (m, 16H, H_4, β-OCH_a, NCH_2CH_2); 3.79-3.63 (m, 16H, H_5, β-OCH_b, $ArCH_2Ar$); 3.60-3.43 (m, 24H, 3CH_2 ethylene glycol chain); 3.22 (t, 8H, J = 6.9 Hz, $OCH_2CH_2CH_3$); 3.05 (t, 8H, J = 7.2 Hz, $COCH_2CH_2$triazole); 2.71 (t, 8H, J = 7.2 Hz, $COCH_2CH_2$triazole); 2.11, 2.08, 2.03, 2.02, 2.01, 1.99, 1.91 (7s, 84H, $COCH_3$); 1.38-1.18 (m, 8H, $OCH_2CH_2CH_3$); 0.64 (t, 12H, J = 7.4 Hz, $OCH_2CH_2CH_3$). ^{13}C NMR (75 MHz, CD_3OD): δ (ppm) 170.9, 170.7, 170.6, 170.5, 170.3, 170.0, 169.9, 169.7 (CH_3CO, CONH); 153.3 (Ar ipso); 146.2 (C_q triazole); 134.2 (Ar ortho); 132.9 (Ar para); 123.0 (CH triazole); 120.8 (Ar meta); 100.6, 100.3 (C_1, C_1'); 76.3 (C_4); 73.1 (C_3); 72.7 (C_5); 72.3 ($OCH_2CH_2CH_3$); 71.7 (C_2); 71.1 (C_3'); 70.3, 70.2, 70.2, 69.9, 69.3, 69.1, 68.8 (C_5', C_2', β-OCH_2, 3CH_2 ethylene glycol chain, NCH_2CH_2,); 67.2 (C_4'); 62.3 (C_6); 60.9 (C_6'); 50.0 (NCH_2); 38.0 ($ArCH_2Ar$); 35.9 ($COCH_2CH_2$triazole); 22.5 ($OCH_2CH_2CH_3$); 21.2 ($COCH_2CH_2$triazole); 19.8, 19.5, 19.4, 19.3, 19.1 ($COCH_3$); 9.2 ($OCH_2CH_2CH_3$). HRMS (ESI) m/z: $[M + 3Na]^{3+}$ Calcd for $C_{188}H_{256}N_{16}O_{88}Na_3$ 1404.85751; Found 1404.85695.

General procedure for deacetylation reactions (compounds 1, 2, 3, 4)

Peracetylated glyco-clusters were dissolved in MeOH and drops of a freshly prepared methanol solution of MeONa were added till pH 8–9. The mixture was stirred at room temperature for 1 or 2 hours. The progress of the reaction was monitored via TLC and/or ESIMS. When a precipitate was observed, H_2O was added to help complete solubilisation. Amberlite resin IR 120/H^+ was subsequently added for quenching and gently stirred for 30 min till neutral pH. The resin was then filtered off and the solvent removed under vacuum to give pure product.

Cone-galactosyl-calix[4]arene (1)

Cone-peracetylated-galactosyl-calix[4]arene **9** (30.0 mg, 10.0 μmol) in 4 mL MeOH. Reaction time: 1 hour. TLC (eluent: butanol/acetone/H_2O 35:35:15). Product **1** was obtained as a white solid. Yield: quantitative. ^1H NMR (300 MHz, CD_3OD): δ (ppm) 7.81 (s, 4H, CH triazole); 6.92 (s, 8H, Ar); 4.52 (t, 8H, J = 4.8 Hz, NCH_2); 4.45 (d, 4H, J = 13.2 Hz, *ax*-$ArCH_2Ar$); 4.25 (d, 4H, J = 7.2 Hz, H_1); 4.00-3.91 (m, 4H, β-OCH_a); 3.90-3.78 (m, 20H, H_4, $OCH_2CH_2CH_3$, NCH_2CH_2); 3.77-3.71 (m, 8H, H_{6a}, H_{6b}); 3.70-3.61 (m, 4H, β-OCH_b); 3.60-3.44 (m, 36H, H_2, H_3, H_5, CH_2 ethylene glycol chain); 3.11 (d, 4H, J = 13.2 Hz, *eq*-$ArCH_2Ar$); 3.01 (t, 8H, J = 7.4 Hz, $COCH_2CH_2$triazole); 2.64 (t, 8H, J =7.4 Hz, $COCH_2CH_2$triazole); 2.03-1.87 (m, 8H, $OCH_2CH_2CH_3$); 1.01 (t, 12H, J = 7.4 Hz, $OCH_2CH_2CH_3$). ^{13}C NMR (100 MHz, CD_3OD): δ (ppm) 171.0 (NHCO); 153.0 (Ar ipso); 146.2 (C_q triazole); 134.8 (Ar ortho); 132.3 (Ar para); 123.2 (CH triazole); 120.5 (Ar meta); 103.7 (C_1); 76.8 ($OCH_2CH_2CH_3$); 75.3, 73.5, 71.2 (C_2, C_3, C_4); 70.0 (ethylene glycol chain); 68.9 (C_4, NCH_2CH_2); 68.2 (β-OCH_2); 61.2 (C_6); 50.1 (NCH_2); 35.7 ($COCH_2CH_2$triazole); 30.7 ($ArCH_2Ar$); 22.9 ($OCH_2CH_2CH_3$); 20.9 ($COCH_2CH_2$triazole); 9.4 ($OCH_2CH_2CH_3$). HRMS (ESI) m/z: $[M + 3Na]^{3+}$ Calcd for $C_{108}H_{160}N_{16}O_{40}Na_3$ 796.68848; Found 796.68798.

Cone-galactosyl-calix[4]arene (2)

Cone-peracetylated-galactosyl-calix[4]arene **13** (60.0 mg, 19.6 μmol) in 10 mL MeOH. Reaction time: 1 hour. TLC (eluent: AcOEt/isopropanol/H_2O 5:2:1). Product **2** was obtained as a white solid. Yield: quantitative. ^1H NMR (300 MHz, CD_3OD): δ (ppm) 9.70 (bs, 4H, NHCO); 8.04 (bs, 4H, CH triazole); 7.01 (s, 8H, Ar); 5.22 (bs, 8H, $COCH_2$triazole); 4.62 (bs, 8H, OCH_2triazole); 4.45 (d, 4H, J = 12.6 Hz, *ax*-$ArCH_2Ar$); 4.24 (d, 4H, J = 7.2 Hz, H_1); 4.02-3.92 (m, 4H, β-OCH_a); 3.91-3.80 (m, 12H, H_4, $OCH_2CH_2CH_3$); 3.79-3.56 (m, 52H, H_{6a}, H_{6b}, β-OCH_b, CH_2 ethylene glycol chain); 3.55-3.44 (m, 12H, H_2, H_3, H_5); 3.12 (d, 4H, J = 12.6 Hz, *eq*-$ArCH_2Ar$); 2.01-1.87 (m, 8H, $OCH_2CH_2CH_3$); 1.02 (t, 12H, J = 7.3 Hz, $OCH_2CH_2CH_3$). ^{13}C NMR (75 MHz, CD_3OD): δ (ppm) 164.0 (NHCO); 153.2 (Ar ipso); 144.5 (C_q triazole); 135.0 (Ar ortho); 132.0 (Ar para); 125.9 (CH triazole); 120.1 (Ar meta); 103.7 (C_1); 76.9 ($OCH_2CH_2CH_3$); 75.3, 73.5 (C_3, C_5), 71.2 (C_2); 70.1, 69.4 (ethylene glycol chain); 68.9 (C_4); 68.2 (β-OCH_2); 63.5 (OCH_2triazole); 61.2 (C_6); 52.3($COCH_2$triazole); 30.7 ($ArCH_2Ar$); 23.0 ($OCH_2CH_2CH_3$); 9.4 ($OCH_2CH_2CH_3$). HRMS (ESI) m/z: $[M + 3Na]^{3+}$ Calcd for $C_{108}H_{160}N_{16}O_{44}Na_3$ 818.01503; Found 818.01577.

***Cone*-lactosyl-calix[4]arene (3)**

Cone-peracetylated-lactosyl-calix[4]arene **16** (46.0 mg, 11.1 µmol) in 10 mL MeOH. Reaction time: 2 hours. Product **3** was obtained as a white solid. Yield: 73%. ^{1}H NMR (300 MHz, CD_3OD): δ (ppm) 7.82 (s, 4H, CH triazole); 6.93 (bs, 4H, Ar); 6.91 (bs, 4H, Ar); 4.54 (t, 8H, J = 4.5 Hz, NCH_2); 4.45 (d, 4H, J = 13.0 Hz, *ax*-ArCH_2Ar); 4.40-4.29 (m, 8H, $H_{1,}$ H_1'); 4.00-3.64 (m, 44H, H_4', H_{6a}, H_{6b}, H_6'$_a$, H_6'$_b$, β-OCH_2, NCH_2CH_2, O$CH_2$$CH_2CH_3$); 3.63-3.39 (m, 48H, H_2', H_3', H_5', H_3, H_4, H_5, 3CH_2 ethylene glycol chain); 3.26 (t, 4H, J = 8.3 Hz, H_2); 3.13 (d, 4H, J = 13.0, *eq*-ArCH_2Ar); 3.02 (t, 8H, J = 7.2 Hz, $COCH_2CC_2$triazole); 2.65 (t, 8H, J = 7.2 Hz, $COCC_2CH_2$triazole); 2.08-1.85 (m, 8H, $OCH_2CC_2CH_3$); 1.02 (t, 12H, J = 7.4 Hz, $OCH_2CH_2CC_3$). ^{13}C NMR (75 MHz, CD_3OD): δ (ppm) 170.9 (CONH); 152.9 (Ar ipso); 146.2 (C_q triazole); 134.8 (Ar ortho); 132.4 (Ar para); 123.1 (CH triazole); 120.4 (Ar meta); 103.7 (C_1'); 102.9 (C_1); 79.3 (C_4); 76.8 (O$CH_2CH_2CH_3$); 75.7, 75.1, 74.9, 73.4, 73.3, 71.1 (C_2, C_3, C_5, C_2'$_,$ C_3', C_5'); 70.0 (3CH_2 ethylene glycol chain); 69.1 (NCH_2CH_2); 68.9 (C_4'); 68.4 (β-OCH_2); 61.1, 60.6 (C_6, C_6'); 49.9 (NCH_2); 35.7 (COCH_2CH_2triazole); 30.7 (ArCH_2Ar); 23.0 ($OCH_2CH_2CH_3$); 20.9 ($COCH_2CH_2$triazole); 9.5 ($OCH_2CH_2CH_3$). HRMS (ESI) m/z: $[M + 3Na]^{3+}$ Calcd for $C_{132}H_{200}N_{16}O_{60}Na_3$ 1012.75891; Found 1012.75902.

***Alt*-lactosyl-calix[4]arene (4)**

Alt-peracetylated-lactosyl-calix[4]arene **19** (23.0 mg, 5.5 µmol) in 5 mL MeOH. Reaction time: 2 hours. Product **4** was obtained as a white solid. Yield: 71%. ^{1}H NMR (400 MHz, CD_3OD): δ (ppm) 8.07 (bs, 4H, CH triazole); 7.35 (bs, 8H, Ar); 4.64 (t, 8H, J = 4.7 Hz, NCH_2); 4.37 (d, 4H, J = 7.7 Hz, H_1'); 4.35 (d, 4H, J = 8.0 Hz, H_1); 4.04-3.95 (m, 4H, β-OCH_a); 3.94-3.68 (m, 40H, H_4', H_{6a}, H_{6b}, H_6'$_a$, H_6'$_b$, NCH_2CC_2, β-OCH_b, ArCC_2Ar); 3.67-3.39 (m, 48H, 3CH_2 ethylene glycol chain, H_2', H_3', H_5', H_3, H_4, H_5); 3.31-3,21 (m, 12H, H_2, $OCC_2CH_2CH_3$); 3.13 (t, 8H, J = 7.1 Hz, $COCH_2CC_2$triazole); 2.79 (t, 8H, J = 7.1 Hz, $COCC_2CH_2$triazole); 1.38-1.19 (m, 8H, $OCH_2CC_2CH_3$); 0.67 (t, 12H, J = 7.4, $OCH_2CH_2CC_3$). ^{13}C NMR (100 MHz, CD_3OD): δ(ppm) 170.7 (CONH); 153.3 (Ar ipso); 145.7 (C_q triazole); 134.3 (Ar ortho); 132.8 (Ar para); 124.1 (CH triazole); 120.9 (Ar meta); 103.8 (C_1'); 102.9 (C_1); 79.4 (C_4); 75.7, 75.1, 74.9, 73.4, 71.2, (C_3, C_5, C_2', C_3', C_5'); 73.3 (C_2); 72.3 (O$CH_2CH_2CH_3$); 70.0 (H_2, 3CH_2 ethylene glycol chain); 68.9, 68.8 (NCH_2CH_2, C_4'); 68.4 (β-OCH_2); 61.1, 60.6 (C_6, C_6'); 50.6 (NCH_2); 38.0 (ArCH_2Ar); 35.4 (COCH_2CH_2triazole); 22.5 ($OCH_2CH_2CH_3$); 20.7 ($COCH_2CH_2$triazole); 9.2 ($OCH_2CH_2CH_3$). HRMS (ESI) m/z: $[M + 3Na]^{3+}$ Calcd for $C_{132}H_{200}N_{16}O_{60}Na_3$ 1012.75891; Found 1012.75923.

Expression and purification of Gal-3

E. coli strain BL21 expressing full length Gal-3 fused to a N-terminal His_6 tag was grown at 30 °C in LB containing zeocin (25 μg/mL) for 16 h. The culture was diluted 1:1000 in fresh medium and grown again. Reached the mid-logarithmic phase (OD_{600nm} = 0.7), the expression of His_6 tagged Gal-3 was induced adding 1 mM isopropyl β-D-1-thiogalactopyranoside (IPTG, Sigma-Aldrich, St. Louis, MO, USA) for 3.5 h at 30 °C. Cells were then harvested by centrifugation (4,000 rpm, 15 min) and resuspended in lysis buffer (50mM phosphate, 300 mM NaCl, 10 mM imidazole) containing protease inhibitor cocktail (Sigma-Aldrich, St. Louis, MO, USA). Cells were disrupted by one cicle of French press (*One Shot* Model Cell Disrupter, Constant Systems LTD, Low March, Daventry, UK) at 25,000 psi and the crude extract was centrifuged at 5000 rpm for 15 min. The cleared solution was loaded on IMAC resin (Qiagen, Hilden, Germany), washed with 10 mL of lysis buffer and eluted 5 times in elution buffer (50 mM phosphate, 300 mM NaCl, 150 mM imidazole, pH 8).
Protein purification was monitored by SDS-PAGE 12% and the protein concentration was determined by a Coomassie (Bradford) assay kit (Sigma-Aldrich, St. Louis, MO, USA), using bovine serum albumin as a standard.

Gal-3/Glycocalixarene binding experiments

SPR (surface plasmon resonance) binding experiments were performed using a BIAcore X system. Carboxymethylated dextran pre-immobilized with nitrilotriacetic acid (NTA) chip (GE Healthcare, Uppsala, Sweden) was used to immobilize His-tagged Gal-3 (200 nM) via Ni^{2+}/NTA chelation. NTA sensor chip was pre-activated with $NiCl_2$ (500 mM) at a flow rate of 30 μL min^{-1}. Surface regeneration was accomplished by multiple injections of Hepes (10 mM, pH 8.2), NaCl (150 mM), EDTA (350 mM), Tween-20 (0.005%) at a flow rate of 5–10 μL min^{-1} and then by $NiCl_2$ at a flow rate of 30 μL min^{-1}. The 1 mM solutions of compounds **1**, **3** and **4** in Hepes (10 mM, pH 7.4), NaCl (150 mM), EDTA (50 mM), and Tween-20 (0.005%) were injected at a flow rate of 5 μL min^{-1}.

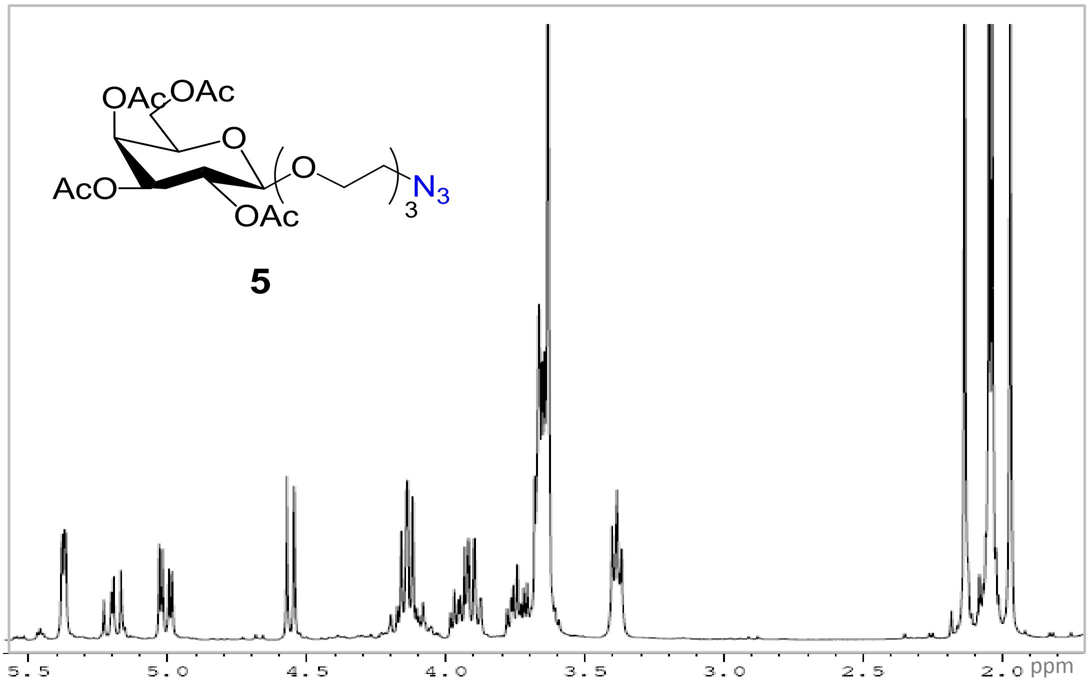

Figure S1: ^{1}H NMR spectrum (300 MHz, $CDCl_3$) of compound **5**.

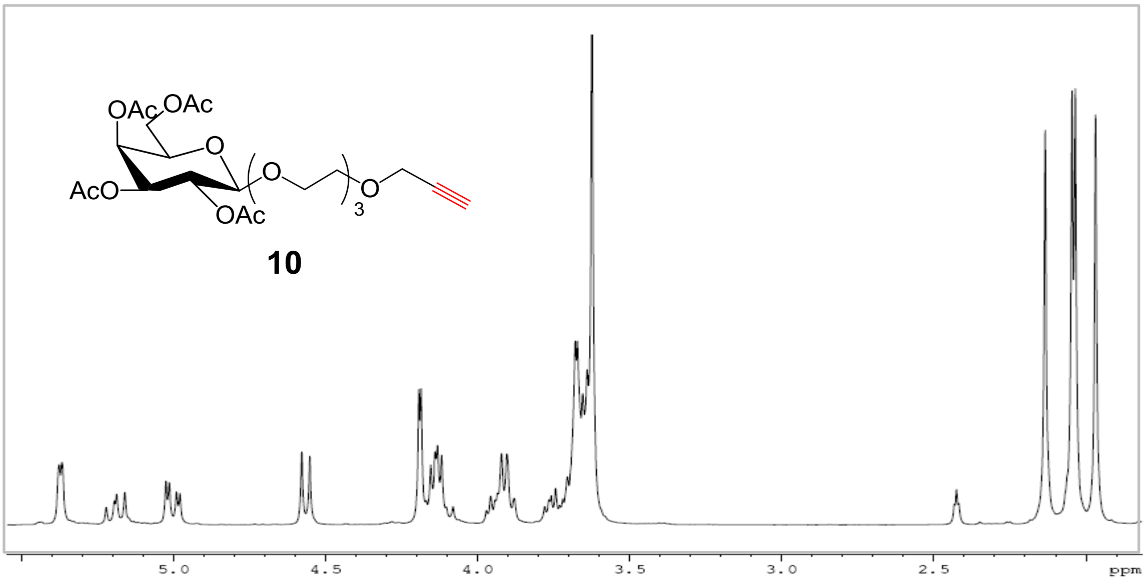

Figure S2: ^{1}H NMR spectrum (400 MHz, $CDCl_3$) of compound **10**.

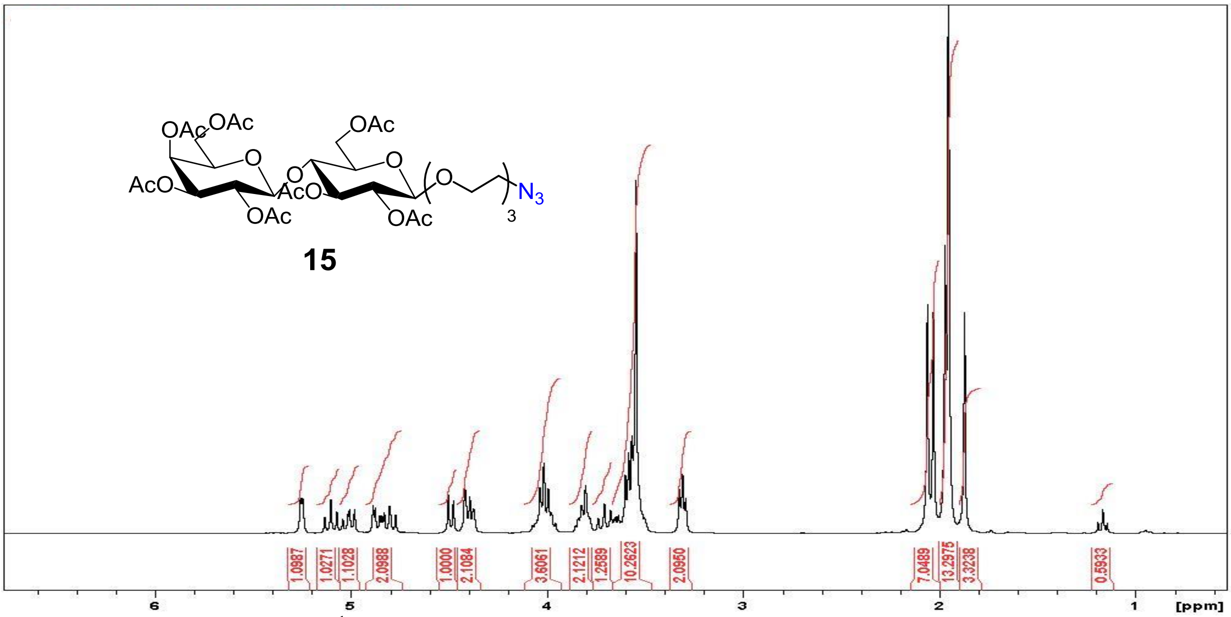

Figure S3: ^{1}H NMR spectrum (300 MHz, $CDCl_3$) of compound **15**.

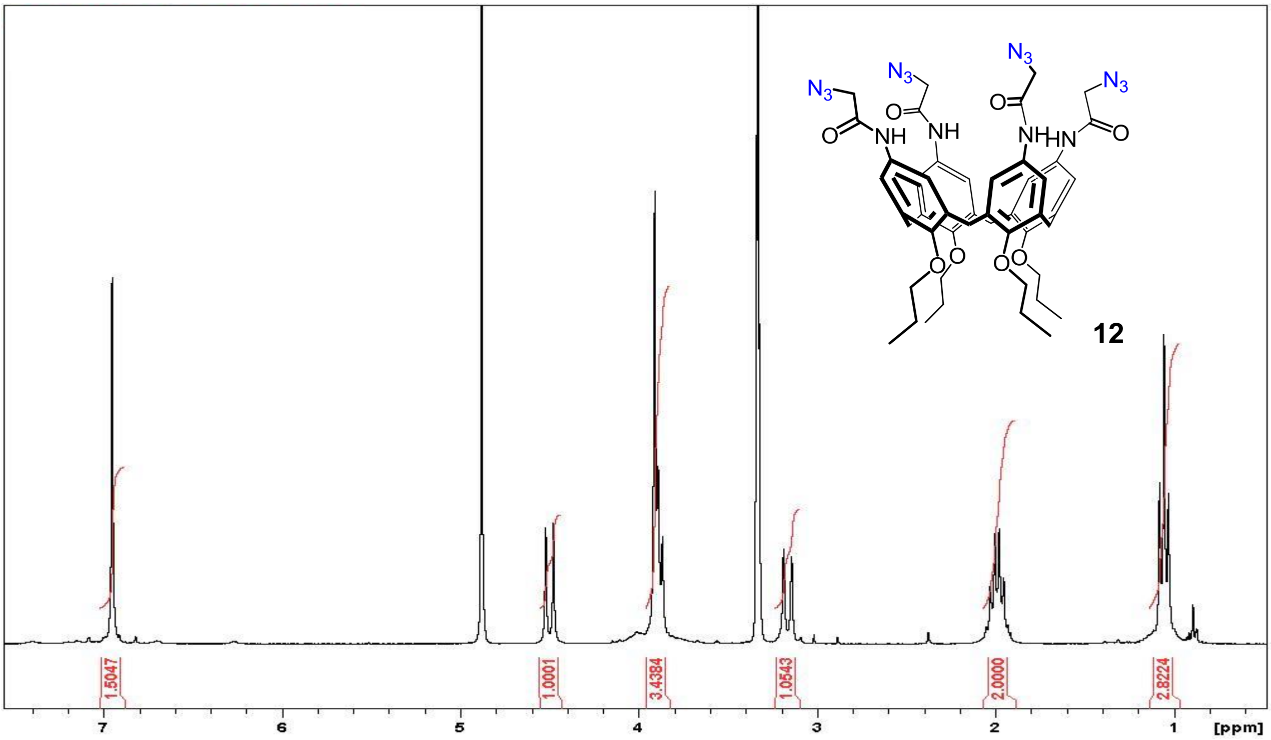

Figure S4: ^{1}H NMR spectrum (300MHz, CD_3OD) of compound **12**.

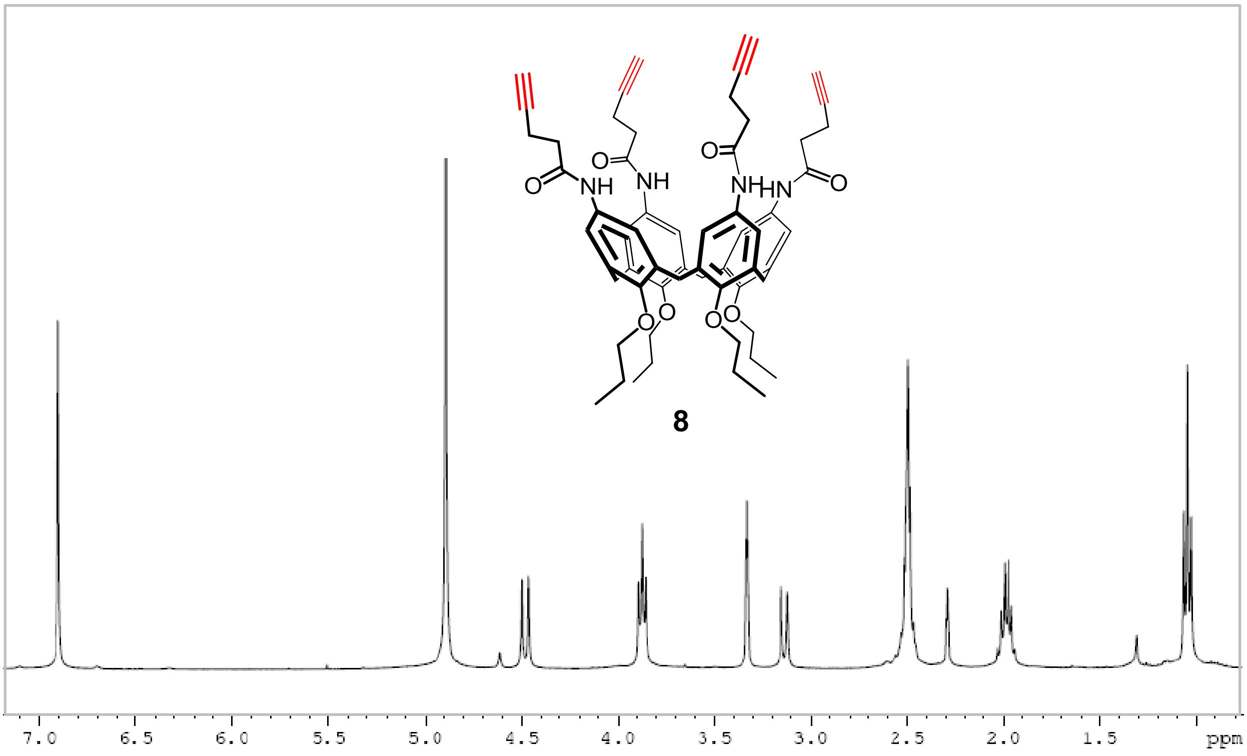

Figure S5: ^{1}H NMR spectrum (400 MHz, CD_3OD) of compound **8**.

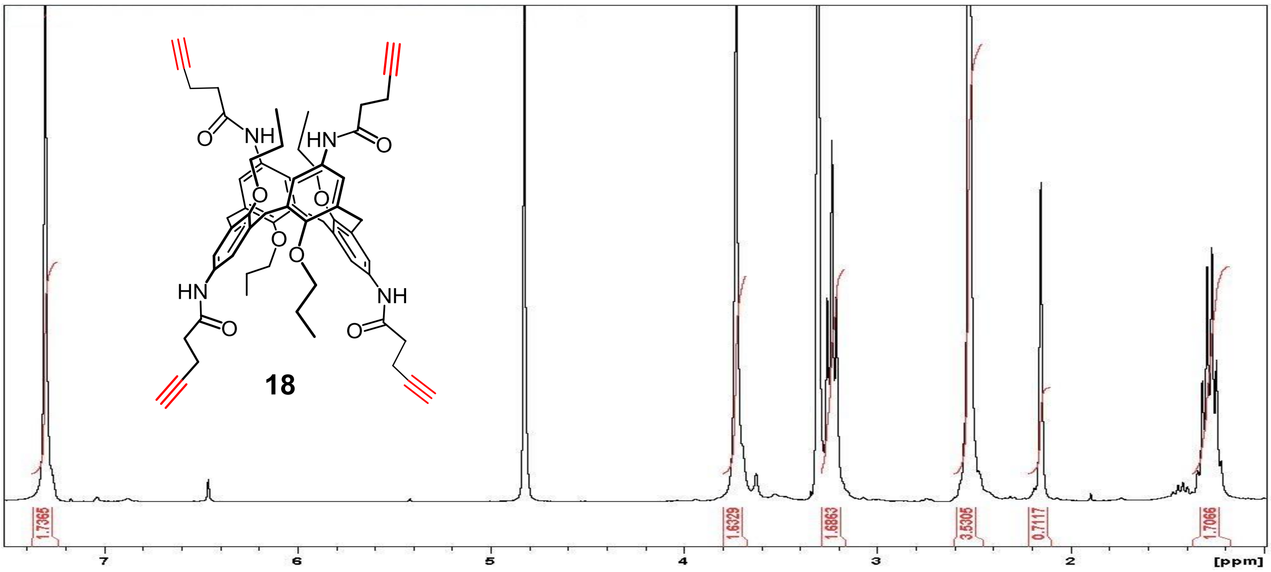

Figure S6: ^{1}H NMR spectrum (300 MHz, $CD_3OD/CDCl_3$ 9:1) of compound **18**.

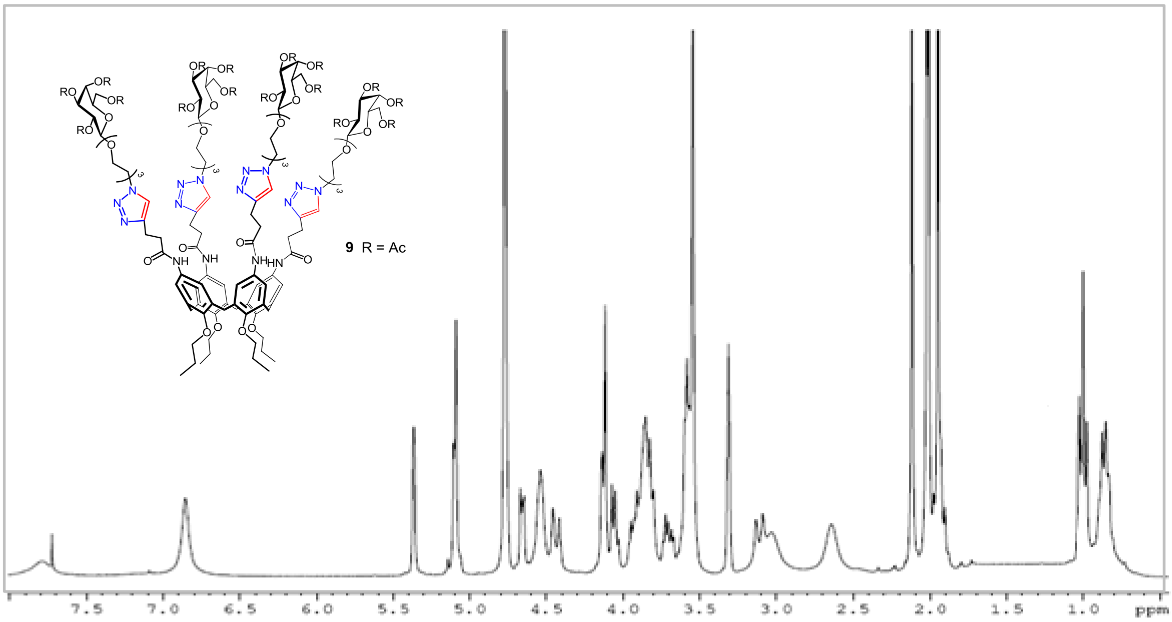

Figure S7: ^{1}H NMR spectrum (400 MHz, $CD_3OD/CDCl_3$ 4:1) of compound ***9***.

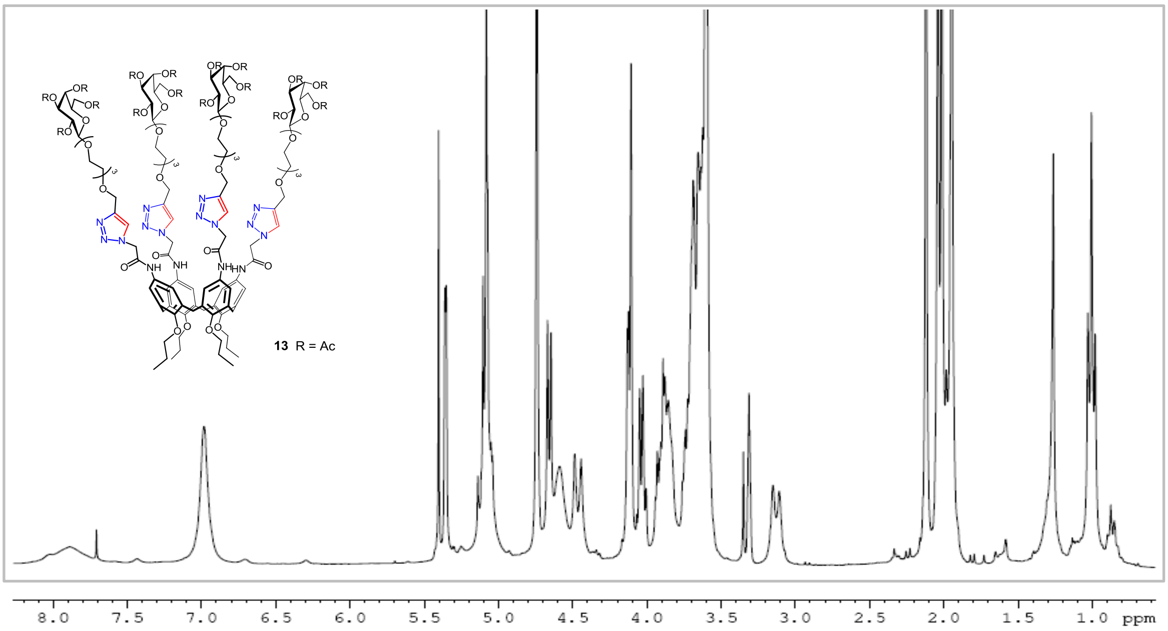

Figure S8: ^{1}H NMR spectrum (300 MHz, $CD_3OD/CDCl_3$ 3:1) of compound **13**.

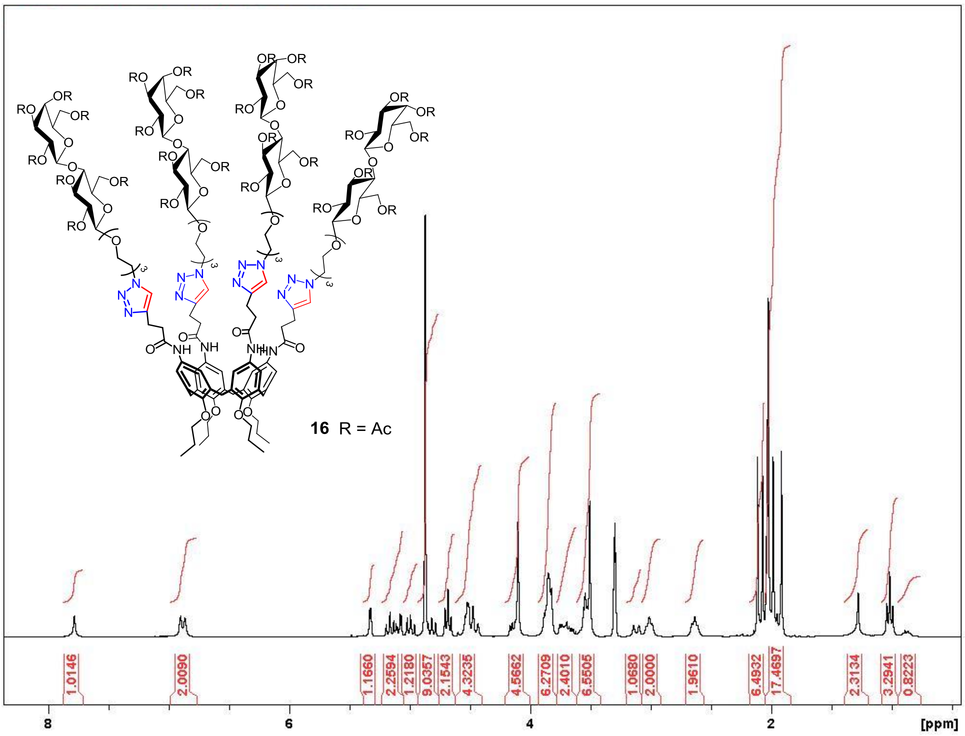

Figure S9: ^{1}H NMR spectrum (300 MHz, CD_3OD) of compound **16**.

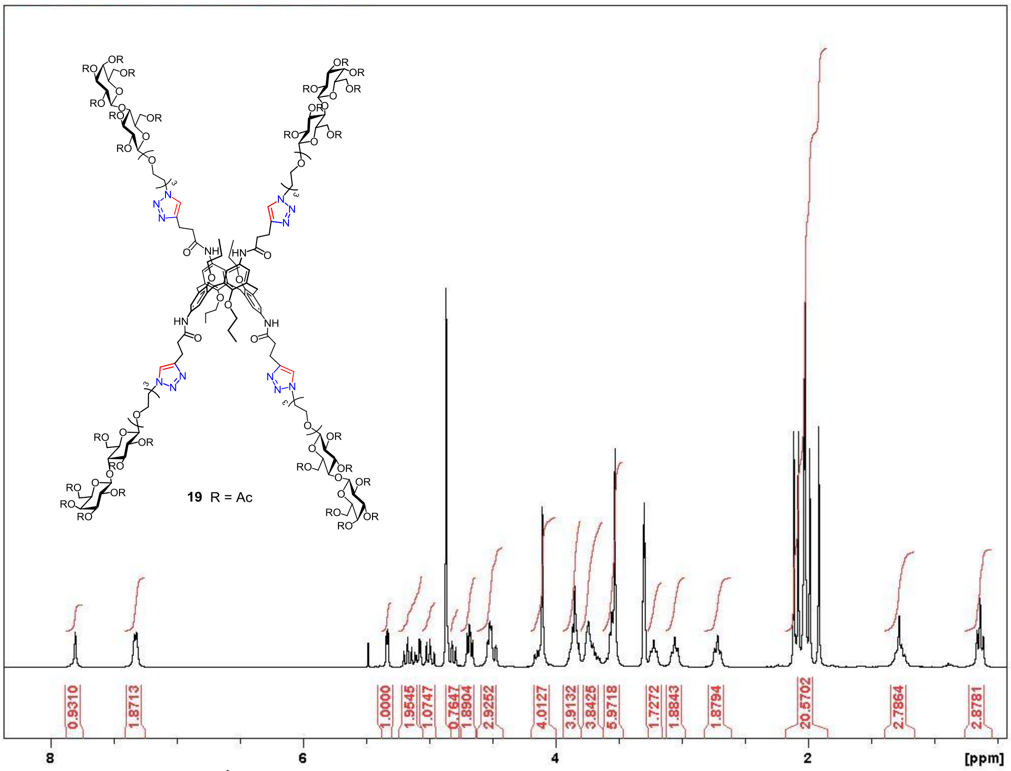

Figure S10: ^{1}H NMR spectrum (300 MHz, CD_3OD) of compound **19**.

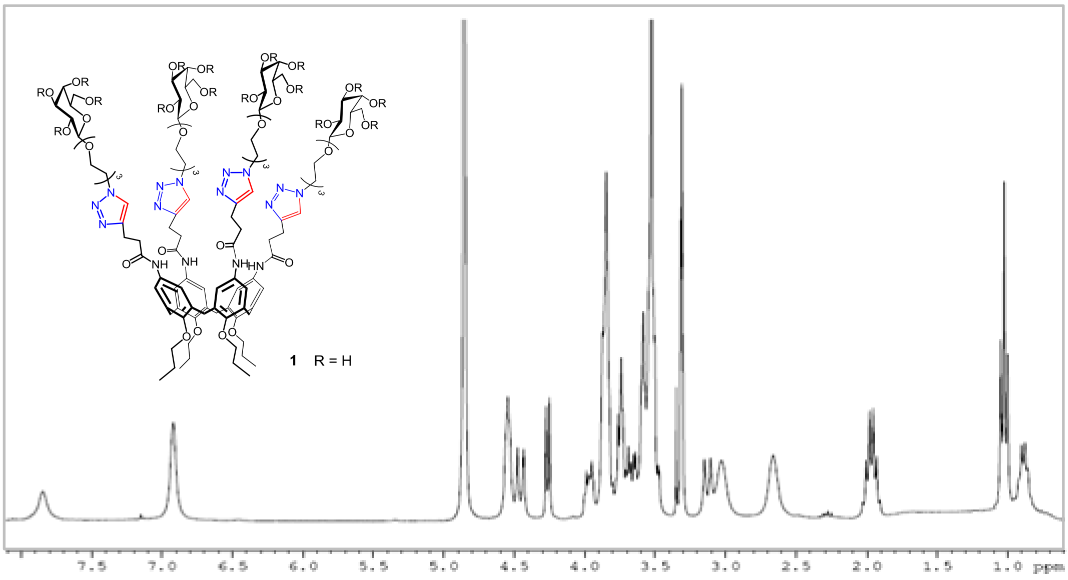

Figure S11: ^{1}H NMR spectrum (300 MHz, CD_3OD) of compound **1**.

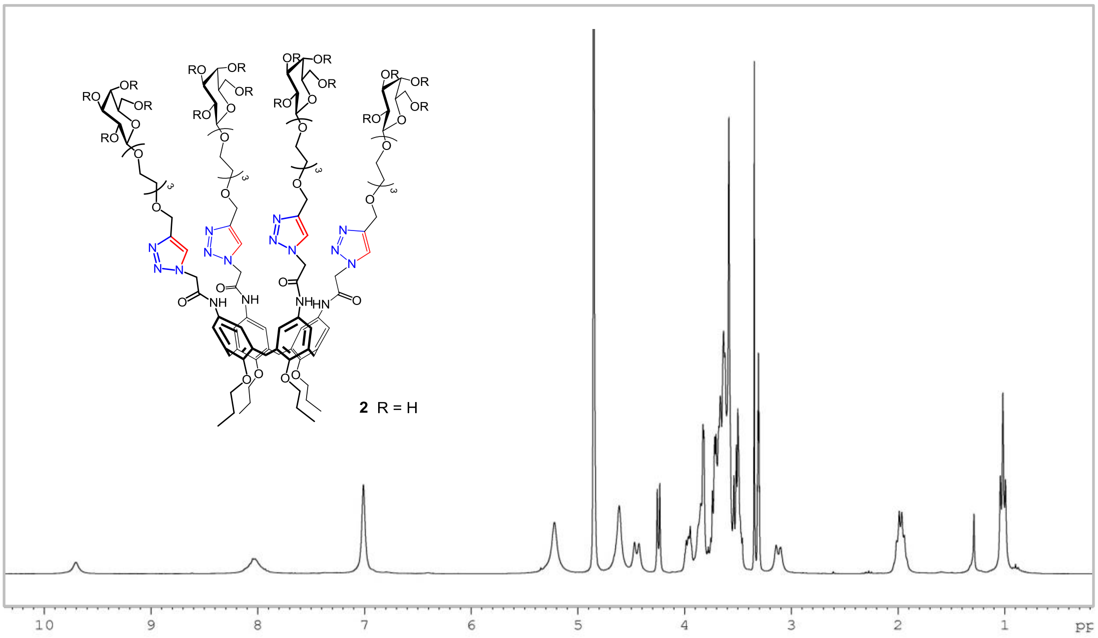

Figure S12: ^{1}H NMR spectrum (300 MHz, CD_3OD) of compound **2**.

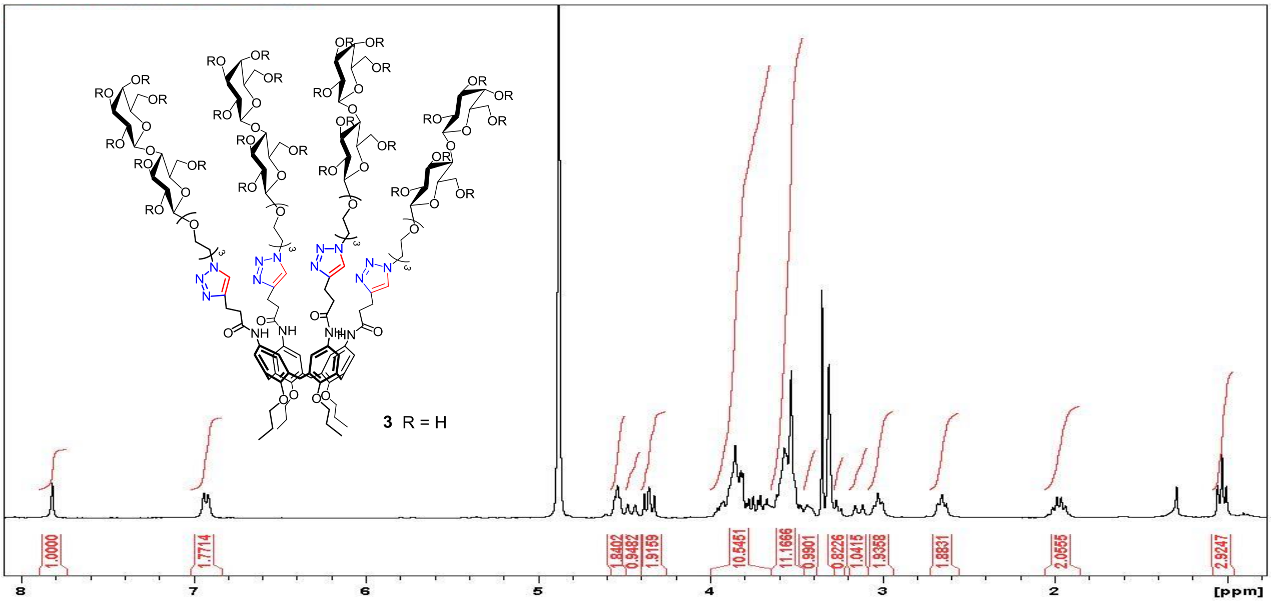

Figure S13: ^{1}H NMR spectrum (300 MHz, CD_3OD) of compound **3**.

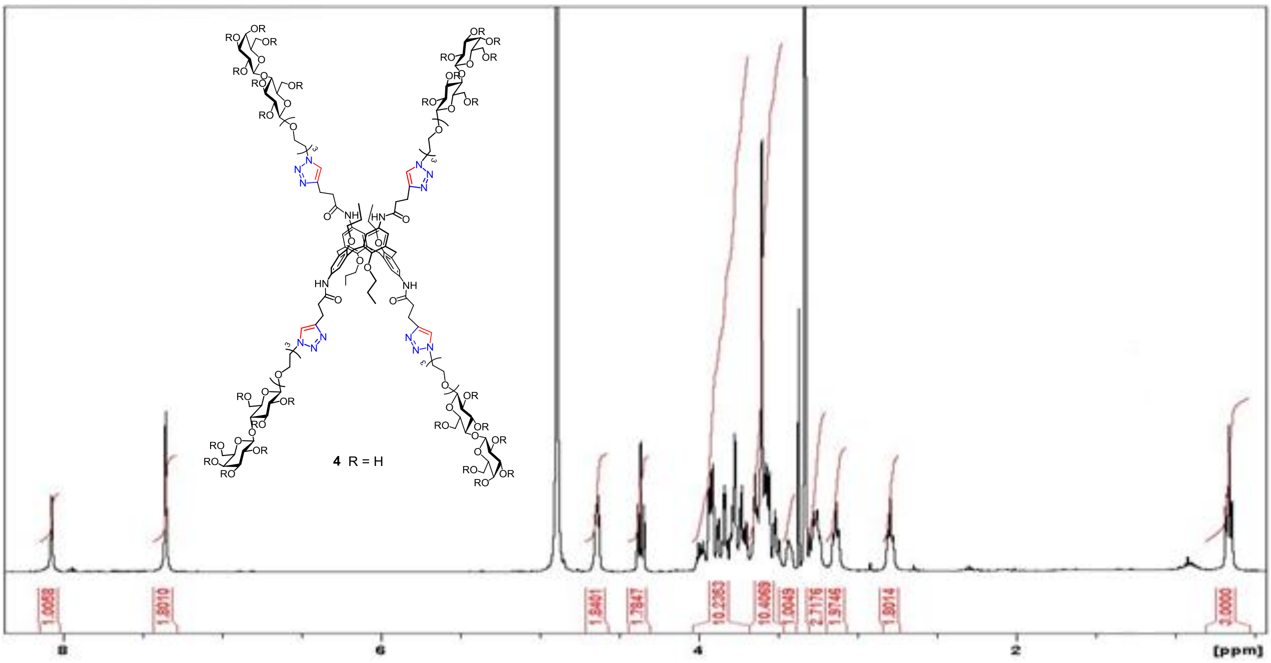

Figure S14: ^{1}H NMR spectrum (400 MHz, CD_3OD) of compound **4**.

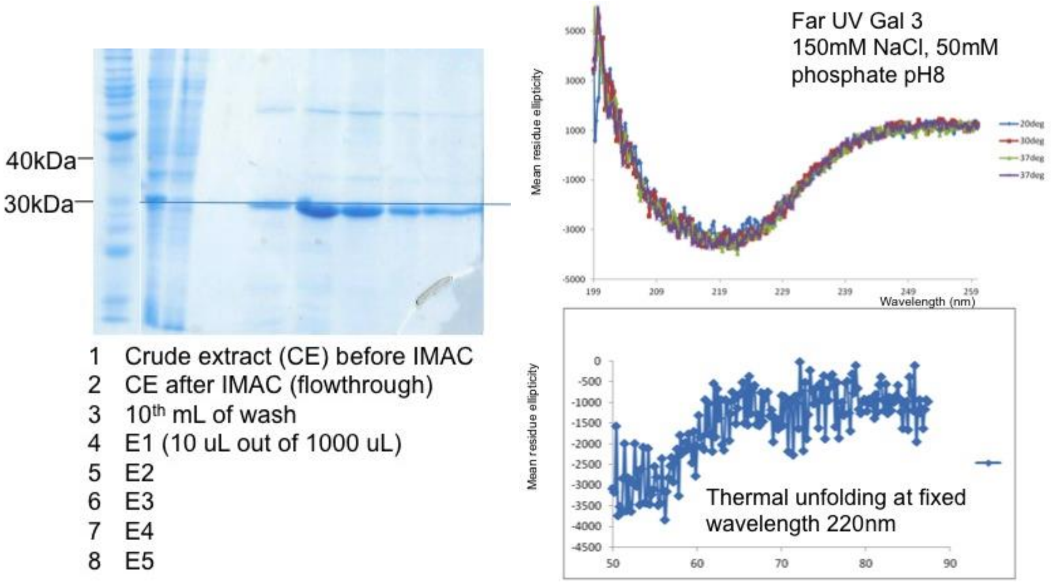

Figure S15: electrophoresis gel of Gal-3 (left), columns 1 e 2 are the crude extract, fractions 4-7 are different eluates of IMAC column with the band of pure Gal-3 protein (approximate molecular weight 30 kD). On the right: circular dichroism spectrum and thermal unfolding of purified Gal-3.

References

[1]. Bouillon, C.; Meyer, A.; Vidal, S.; Jochum, A.; Chevolot, Y.; Cloarec, J. P.; Praly, J. P.; Vasseur, J. J.; Morvan, F. *J. Org. Chem.* **2006**, *71*, 4700-4702.

[2]. Michel, O.; Ravoo, B. J. *Langmuir* **2008**, *24*, 12116-12118.

[3]. Sansone, F.; Chierici, E.; Casnati, A.; Ungaro, R. *Org. Biomol. Chem.* **2003**, *1*, 1802-1809.

[4]. Alyapyshev, M. Y.; Babain, V. A.; Boyko, V. I.; Eliseev, I. I.; Kirsanov, D. O.; Klimchuk, O. V.; Legin, A. V.; Mikhailina, E. S.; Rodik, R. V.; Smirnov, I. V. *J. Inclusion Phenom. Macrocyclic Chem.* **2010**, *67*, 117-126.

[5]. Kato, H.; Uzawa, H.; Nagatsuka, T.; Kondo, S.; Sato, K.; Ohsawa, I.; Kanamori-Kataoka, M.; Takei, Y.; Ota, S.; Furuno, M.; Dohi, H.; Nishida, Y.; Seto, Y. *Carbohydr. Res.* **2011**, *346*, 1820-1826.

[6]. Dudic, M.; Colombo, A.; Sansone, F.; Casnati, A.; Donofrio, G.; Ungaro, R. *Tetrahedron* **2004**, *60*, 11613-11618.

[7]. Sansone, F.; Dudic, M.; Donofrio, G.; Rivetti, C.; Baldini, L.; Casnati, A.; Cellai, S.; Ungaro, R. *J. Am. Chem. Soc.* **2006**, *128*, 14528-14536.
